# Phylogenetic and experimental characterization of an acyl-ACP thioesterase family reveals significant diversity in enzymatic specificity and activity

**DOI:** 10.1186/1471-2091-12-44

**Published:** 2011-08-10

**Authors:** Fuyuan Jing, David C Cantu, Jarmila Tvaruzkova, Jay P Chipman, Basil J Nikolau, Marna D Yandeau-Nelson, Peter J Reilly

**Affiliations:** 1 Department of Biochemistry, Biophysics, and Molecular Biology, Biorenewables Research Laboratory Building, Iowa State University, Ames, Iowa 50011-3270, USA; 2 Department of Chemical and Biological Engineering, 2114 Sweeney Hall, Iowa State University, Ames, Iowa 50011-2230, USA

## Abstract

**Background:**

Acyl-acyl carrier protein thioesterases (acyl-ACP TEs) catalyze the hydrolysis of the thioester bond that links the acyl chain to the sulfhydryl group of the phosphopantetheine prosthetic group of ACP. This reaction terminates acyl chain elongation of fatty acid biosynthesis, and in plant seeds it is the biochemical determinant of the fatty acid compositions of storage lipids.

**Results:**

To explore acyl-ACP TE diversity and to identify novel acyl ACP-TEs, 31 acyl-ACP TEs from wide-ranging phylogenetic sources were characterized to ascertain their *in vivo *activities and substrate specificities. These acyl-ACP TEs were chosen by two different approaches: 1) 24 TEs were selected from public databases on the basis of phylogenetic analysis and fatty acid profile knowledge of their source organisms; and 2) seven TEs were molecularly cloned from oil palm (*Elaeis guineensis*), coconut (*Cocos nucifera*) and *Cuphea viscosissima*, organisms that produce medium-chain and short-chain fatty acids in their seeds. The *in vivo *substrate specificities of the acyl-ACP TEs were determined in *E. coli*. Based on their specificities, these enzymes were clustered into three classes: 1) Class I acyl-ACP TEs act primarily on 14- and 16-carbon acyl-ACP substrates; 2) Class II acyl-ACP TEs have broad substrate specificities, with major activities toward 8- and 14-carbon acyl-ACP substrates; and 3) Class III acyl-ACP TEs act predominantly on 8-carbon acyl-ACPs. Several novel acyl-ACP TEs act on short-chain and unsaturated acyl-ACP or 3-ketoacyl-ACP substrates, indicating the diversity of enzymatic specificity in this enzyme family.

**Conclusion:**

These acyl-ACP TEs can potentially be used to diversify the fatty acid biosynthesis pathway to produce novel fatty acids.

## Background

*De novo *fatty acid biosynthesis can be considered an iterative "polymerization" process, commonly primed with the acetyl moiety from acetyl-CoA and with iterative chain extension occurring by reaction with malonyl-ACP. In most organisms this process optimally produces 16- and 18-carbon (C16 and C18) fatty acids. The enzyme that determines fatty acid chain length is acyl-acyl carrier protein thioesterase (acyl-ACP TE). This enzyme catalyzes the terminal reaction of fatty acid biosynthesis, acyl-ACP thioester bond hydrolysis to release a free fatty acid and ACP.

In discrete phyla and/or tissues of specific organisms (primarily higher plant seeds), thioester hydrolysis optimally produces medium-chain (C8-C14) fatty acids (MCFAs), which have wide industrial applications (e.g., producing detergents, lubricants, cosmetics, and pharmaceuticals) [1]. TEs that specifically hydrolyze medium-chain acyl-ACP substrates have been studied widely [1-3]. Short-chain fatty acids (SCFAs; e.g. butanoic acid and hexanoic acid) have more recently gained importance as potential biorenewable chemicals that could be derived from the fatty acid biosynthesis pathway [4]. As a critical acyl chain termination enzyme, acyl-ACP TEs with desired substrate specificities are therefore important for engineering this pathway.

To date, dozens of acyl-ACP TEs have been functionally characterized and sorted into two classes, FatA and FatB [5]. FatA-class TEs act on long-chain acyl-ACPs, preferentially on oleoyl-ACP [5-8], while FatB-class TEs preferably hydrolyze acyl-ACPs with saturated fatty acyl chains [5]. The archetypical FatB-class TE was isolated from the developing seeds of California bay (*Umbellularia californica*). This enzyme is specific for 12:0-ACP, and it plays a critical role in MCFA production [2,9]. This discovery spurred isolation of additional MCFA-specific TEs from *Cuphea *[1,10,11], *Arabidopsis thaliana *[12], *Myristica fragrans *(nutmeg) [13], and *Ulmus americana *(elm) [13].

Recently, TEs obtained from public databases were classified into 23 families based on sequence and three-dimensional structure similarity [14]. These TEs were defined as enzymes that can hydrolyze any thioester bond irrespective of the chemical nature of the carboxylic acid and thiol molecules that constitute the substrates of these enzymes. The TE sequences are collected in the constantly updated ThYme database [15]. Of these 23 families, Family TE14 contains plant and bacterial acyl-ACP TEs involved in Type II fatty acid synthesis, whose reactions are catalyzed by discrete monofunctional enzymes. When this study was conducted (summer and fall 2010), Family TE14 contained 360 unique sequences, but only ~7% of these sequences, all of which were FatA and FatB TEs from higher plants, had been functionally characterized. The remaining ~220 bacterial acyl-ACP TEs were mostly generated from genomic sequencing projects and had never been functionally characterized.

Here we report the results of a two-pronged approach to identify acyl-ACP TEs with novel substrate specificities, which potentially could allow researchers to better infer biochemical properties of closely related sequences. This strategy includes the functional characterization of diverse acyl-ACP TEs 1) rationally chosen based on phylogenetic classification of the enzymes and 2) isolated from organisms that are known to produce MCFAs and SCFAs. Functional characterization of 31 acyl-ACP TEs from diverse organisms led to the discovery that several novel TEs can be used to produce short-chain and unsaturated fatty acids as well as methylketones.

## Experimental Procedures

### Phylogenetic analyses

Sequences from Family TE14 [14] in the ThYme database were downloaded from the GenBank [16] and UniProt [17] databases. Fragments and incomplete sequences were removed, yielding 360 acyl-ACP TE sequences. A multiple sequence alignment (MSA) was generated from catalytic domains of these sequences using MUSCLE 3.6 [18] with default parameters. An unrooted phylogenetic tree based on the MSA was built using Molecular Evolutionary Genetics Analysis 4 (MEGA4) [19]. The minimum evolution algorithm was used due to its high effectiveness with large data sets [20], gaps were subjected to pairwise deletion, and an amino acid Jones-Taylor-Thornton (JTT) [21] distance model was chosen. The phylogenetic tree was further verified by a bootstrap test with 1000 replicates. The bootstrapped consensus tree was qualitatively analyzed and broken into apparent subfamilies. Statistical analysis was conducted to show that all sequences within a subfamily were more closely related to each other than to sequences in other subfamilies. Based on the MSA, JTT distances between all sequences were calculated and arranged into a *j *× *j *matrix, where *j *is the total number of sequences. Inter-subfamily distances and variances were determined using this matrix. For each apparent subfamily, a smaller *k *× *k *matrix, where *k *is the number of sequences in a given subfamily, was calculated. From this, intra-subfamily mean distances and variances were determined. These values were applied to the following equation to determine *z*:

where  and  are the inter- and intra-subfamily mean JTT distances, *n_ij_, n_ii_*, and *n_jj _*are the total number of taxa used for each  value, and  and  are the pooled inter- and intra-subfamily variances [22].

A *z*-value > 3.3 between two subfamilies shows that the difference between them is statistically significant to *p *< 0.001. If a *z*-value between two apparent subfamilies were < 3.3, alternative apparent subfamilies were chosen and/or individual sequences were removed, and the statistical calculations were repeated. Subfamilies were finally defined with a phylogenetic tree in which all *z*-values exceeded 3.3, sometimes leaving some sequences outside any subfamily (i.e. non-grouped sequences).

All sequences within individual subfamilies were aligned using MUSCLE 3.6, and rooted phylogenetic trees were built in MEGA4 with the same tree and bootstrap parameters as described above. A few sequences from another subfamily (that with the highest *z*-value) were chosen to root individual subfamily trees.

### DNA synthesis

cDNA sequences encoding acyl-ACP TEs were codon-optimized for expression in *E. coli *using the OptimumGene codon optimization program provided by GenScript USA (Piscataway, NJ, USA). Sequences were both synthesized and cloned into vectors by GenScript. *Bam*HI and *Eco*RI restriction sites were added to the 5' and 3' ends of each sequence, and products were cloned into the pUC57 vector.

### Cloning of acyl-ACP TE cDNAs from coconut (*Cocos nucifera*) and *Cuphea viscosissima*

Coconut fruits of different developmental stages were obtained from the USDA-ARS-SHRS National Germplasm Repository (Miami, FL, USA). Seeds of *C. viscosissima *were obtained from the North Central Regional Plant Introduction Station (NCRPIS, Ames, IA, USA). They were treated overnight with 0.1 mM gibberellic acid and then germinated in a growth chamber (Environmental Growth Chambers, Chagrin Falls, OH) with 12 h of illumination at 25°C followed by 12 h of darkness at 15°C. Seedlings were transplanted into soil and cultivated at NCRPIS. Seeds at different developmental stages were collected and flash-frozen in liquid nitrogen.

Acyl-ACP TE cDNAs were cloned from *C. viscosissima *and coconut via a homologous cloning strategy. MSAs of plant TE14 sequences revealed two conserved regions (RYPTWGD and NQHVNNVK), from which two degenerate primers, DP-F3 (5'-AGNTAYCCNACNTGGGGNGA-3') and DP-R3 (5'-TACTTNACRTTRTTNACRTGYTGRTT-3'), were designed. RNA was extracted from endosperm of nearly mature coconuts and immature seeds of *C. viscosissima *using the total RNA (plant) kit (IBI Scientific, Peosta, IA, USA). RNA was reverse-transcribed to cDNA using the SuperScript™ first-strand synthesis system for RT-PCR kit (Invitrogen, Carlsberg, CA, USA). PCR was performed in a 50-μL reaction mixture containing 20 ng cDNA, 1× *Pfx *buffer, 1 mM MgSO_4_, 0.3 mM dNTP, 5.12 μM DP-F3 and DP-R3 primers, and 0.5 U *Pfx *polymerase (Invitrogen) using a cycling program of 94°C for 4 min, 35 cycles of 94°C for 30 s, 52°C for 30 s and 72°C for 45 s, and a final extension step of 72°C for 5 min. The expected ~350-bp products were identified by agarose gel electrophoresis, and their DNA bands were recovered using the QiaQuick gel extraction kit (Qiagen, Valencia, CA, USA) and cloned into the pENTR TOPO TA vector (Invitrogen). Using primers designed from the sequences of the cloned 350-bp fragments, the 5'- and 3'- ends of the cDNAs were obtained using the SMARTer RACE (rapid amplification of the cDNA ends) cDNA amplification kit (Takara Bio, Otsu, Japan).

For each acyl-ACP TE sequence, the full-length cDNA, minus the N-terminal chloroplast transit peptide, was amplified by PCR with primers engineered to introduce *Bam*HI and *Eco*RI restriction sites at the 5'- and 3'-ends, respectively. The PCR-amplified products were digested with *Bam*HI and *Eco*RI and cloned into the corresponding restriction sites of the pUC57 vector, which placed the acyl-ACP TE sequence under the transcriptional control of the *lac*Z promoter. The sequence of each construct was confirmed by sequencing both strands. Confirmed expression vectors of coconut genes were transformed into *E. coli *strain K27, while sequences of *C. viscosissima *acyl-ACP TEs were synthesized after being codon-optimized.

### *In vivo *activity assay

*E. coli *strain K27 contains a mutation in the *fadD *gene impairing β-oxidation of fatty acids, which results in the accumulation of free fatty acids in the growth medium [23,24]. Each TE was expressed in *E. coli *K27, and free fatty acids that accumulated in the medium were extracted and analyzed. Four colonies for each construct were independently cultured in 2 mL LB medium supplemented with 100 mg/L carbicillin in 17-mL culture tubes. When the culture reached an OD_600 _of ~0.7, the growth medium was replaced with 3 mL of M9 minimal medium (47.7 mM Na_2_HPO_4_, 22.1 mM KH_2_PO_4_, 8.6 mM NaCl, 18.7 mM NH_4_Cl, 2 mM MgSO_4_, and 0.1 mM CaCl_2_) supplemented with 0.4% glucose and 100 mg/L carbicillin, and 10 μM isopropyl-β-D-thiogalactopyranoside (IPTG) was added to induce acyl-ACP TE expression. After 40 h of cultivation, cells were pelleted, and free fatty acids in the supernatant were extracted essentially following a previously described method [25,26]. Briefly, 2 mL of culture supernatant was supplemented with 10 μg heptanoic acid (7:0), 10 μg undecanoic acid (11:0), and 20 μg heptadecanoic acid (17:0) (Sigma-Aldrich, St. Louis, MO, USA) as internal standards. The mixture was acidified with 20 μL of 1 M HCl, and 4 mL chloroform-methanol (1:1 vol/vol) was used to recover the fatty acids from the medium. After vortexing for 10 min and centrifuging at 1000 × *g *for 4 min, the lower chloroform phase was transferred to a new tube and evaporated under a stream of N_2 _gas until the samples were concentrated to ~300 μL. Samples (1 μL) were analyzed on an Agilent Technologies (Santa Clara, CA, USA) 6890 Series gas chromatograph (GC) system used with an Agilent 5973 mass selective detector equipped with an Agilent CP-Wax 58 FFAP CB column (25 m × 0.15 mm × 0.39 mm). The GC program followed an initial temperature of 70°C for 2 min, ramped to 150°C at 10°C/min and held for 3 min, ramped to 260°C at 10°C/min, and held for 14 min. Final quantification analysis was performed with AMDIS software (National Institute of Standards and Technology). Determination of C4 to C8, C10 to C12, and > C12 fatty acid concentrations was based on the fatty acid internal standards 7:0, 11:0, and 17:0, respectively. The total concentration of fatty acids produced by each acyl-ACP TE was obtained by subtracting the concentration of fatty acid produced by *E. coli *expressing a control plasmid (pUC57) lacking a TE from that produced by *E. coli *expressing a given acyl-ACP TE sequence from the same vector. The three most abundant fatty acids produced by the control strain were 8:0 (2.0 nmol/mL), 14:0 (3.5 nmol/mL), and 16:0 (3.1 nmol/mL), and their levels were minimal compared to strains expressing acyl-ACP TEs. Compared to GC analyses of fatty acids after derivatization (e.g., methylation or butylation), our GC-MS method uses non-derivatized free fatty acids, which is better optimized for analyzing short-chain fatty acids (e.g., 4:0, 6:0, 8:0, 10:0, 12:0, and 14:0). However, this method may be less sensitive for longer-chain fatty acids (e.g., 18:0 and 18:1).

### Identification of the methylketone 2-tridecanone

Analysis of free fatty acids revealed possible peaks characteristic of 2-tridecanone. To further confirm this identification, retention times and MS spectra of the peaks in each sample were compared to a 2-tridecanone standard (Sigma-Aldrich).

### Statistical cluster analysis

To classify acyl-ACP TEs based on their *in vivo *activities, the fatty acid composition data obtained from the *in vivo *expression of all TE sequences studied herein were used to perform statistical clustering analysis. The distance matrix was calculated using Euclidean distances, and Ward's method [27] was used to perform agglomerative hierarchical clustering. The *p*-values were calculated via multiscale bootstrap resampling with 1000 replicates [28].

## Results

Two complementary approaches were taken to understand the breadth of substrate specificities exhibited by acyl-ACP TEs isolated from different taxa. In the first approach, we used phylogenetic analysis of all Family TE14 members of known or predicted function to strategically choose diverse TE sequences that were then expressed and functionally characterized. In the second approach, previously uncharacterized acyl-ACP TEs were cloned from seeds of plants known to produce seed oils containing SCFAs and MCFAs.

### Phylogenetic analysis and identification of acyl-ACP TEs

**Figure 1:**
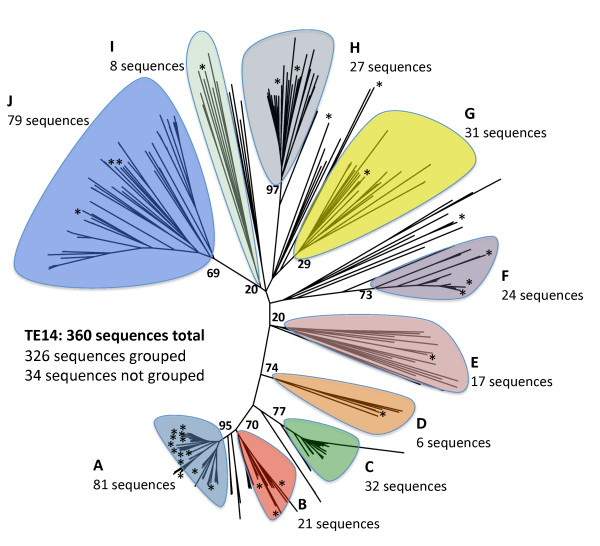
**Unrooted phylogenetic tree of acyl-ACP TEs showing Subfamilies A to J**. Those branches falling outside the shaded areas are non-grouped and therefore are not part of any subfamily. Bootstrap values are shown at each subfamily node. Asterisks denote approximate locations of characterized sequences. A detailed tree for each individual subfamily can be found in Additional files 2, 3, 4, 5, 6, 7, 8, 9, 10, 11, Figures A1-A10.

A total of 360 amino acid sequences belonging to Family TE14 [14] were subjected to phylogenetic analysis and grouped into subfamilies. A subfamily is defined as having at least five sequences from different species, and it must pass the statistical tests described in the experimental procedures. Ten subfamilies met these criteria (Figure 1 and Additional file 1, Table A1), accounting for 326 TE sequences; in addition 34 TE sequences could not be grouped into any of these subfamilies. All *z*-values were > 3.4, ranging from 3.41 to 29.7, and mean distances between different subfamilies were larger than those within subfamilies (Additional file 1, Table A1). Individual trees of each subfamily appear in Additional files 2, 3, 4, 5, 6, 7, 8, 9, 10 and 11, Figures A1 through A10).

Family TE14 contains acyl-ACP TEs that had previously been characterized from plants and classified into two types, FatA and FatB [5]. Of the ten subfamilies identified in this study, Subfamilies A, B, and C are comprised of acyl-ACP TEs found in plants. All experimentally characterized sequences previously classified as FatB acyl-ACP TEs make up ~25% of Subfamily A (Additional file 2, Figure A1), which contains 81 angiosperm-sourced sequences. The coconut and *C. viscosissima *acyl-ACP TEs identified in this study also belong to this subfamily. Subfamily B, which comprises 21 sequences primarily sourced from angiosperms as well as from the moss *Physcomitrella patens *(Additional file 3, Figure A2), represents a potentially novel plant acyl-ACP TE subfamily with no previous experimental or phylogenetic characterization. Plant FatA acyl-ACP TEs, which act on long-chain acyl-ACP molecules, especially oleoyl-ACP [5], belong to the 32-member Subfamily C (Additional file 4, Figure A3). As with Subfamily B, the six green algal sequences from *Chlamydomonas, Ostreococcus*, and *Micromonas *(Additional file 5, Figure A4) that comprise Subfamily D have not been experimentally characterized.

**Figure 2:**
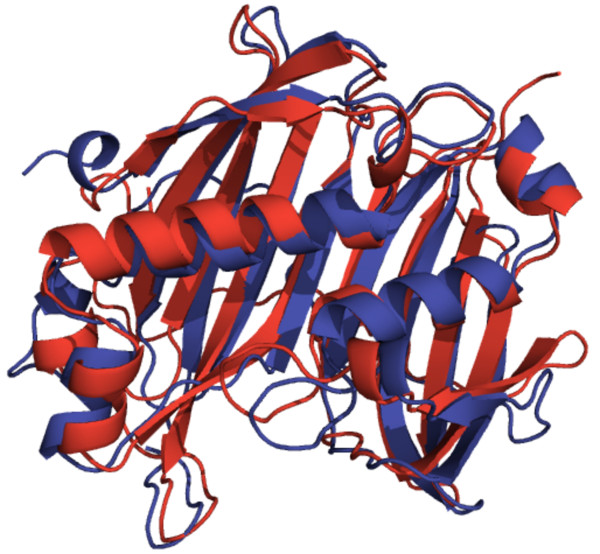
**Superimposed PDB structures**. 2ESS (blue) from *B. thetaiotaomicron *(Subfamily F) and 2OWN (red) from *L. plantarum *(Subfamily J).

Unlike several plant acyl-ACP TEs, no bacterial acyl-ACP TEs had been functionally characterized. A total of 186 bacterial acyl-ACP TE sequences were classified into six subfamilies (Subfamily E-Subfamily J). All 17 acyl-ACP TE sequences from gram-negative bacteria are in Subfamily E (Additional file 6, Figure A5), which includes sequences from halophilic (*Salinibacter *and *Rhodothermus*), sulfate-reducing (*Desulfovibrio, Desulfohalobium*, and *Desulfonatronospira*), chemoorganotrophic (*Spirosoma*), metal-reducing (*Anaeromyxobacter, Geobacter*, and *Pelobacter*), and marine (*Microscilla*) bacteria. Subfamily F consists of 24 sequences, mainly from *Bacteroides *but also from other related bacteria (Additional file 7, Figure A6). Protein Data Bank (PDB) structure 2ESS (Figure 2), obtained from a structural genomic effort, is part of this subfamily. Subfamily G and Subfamily H have 31 and 27 sequences, respectively, primarily from *Clostridium *(Additional files 8 and 9, Figures A7 and A8). Subfamily I is comprised of eight sequences (Additional file 10, Figure A9) from six genera. Gram-positive lactic acid bacteria, almost completely from the genera *Lactobacillus, Enterococcus*, and *Streptococcus*, are part of Subfamily J (79 sequences; Additional file 11, Figure A10). PDB:2OWN (Figure 2), the second bacterial acyl-ACP TE structure obtained from a structural genomic effort, appears in this family. Although the two known Family TE14 crystal structures (PDB:2ESS in Subfamily F and PDB:2OWN in Subfamily J) are from organisms in widely separated subfamilies, they are highly similar, as may be expected since they are members of the same enzyme family (Figure 2).

Some Family TE14 sequences are not grouped into any subfamily because their inclusion decreased *z*-values below acceptable limits. These include two plant and four moss sequences adjacent to Subfamilies A and C, and 28 bacterial sequences more closely related to Subfamilies E to I. No experimental work had previously been done on any of these sequences.

Upon generating the phylogenetic relationships among the 360 acyl-ACP TE sequences predicted or experimentally placed in Family TE14, 25 were chosen for experimental characterization. Of these, the cDNA for 24 was synthesized, while the cDNA of the *Elaeis guineensis *(oil palm) acyl-ACP TE was isolated from a phage cDNA library previously constructed from mRNA isolated from the developing fruit of Indonesian-sourced oil palm.

The selection of acyl-ACP TEs to characterize was based upon the primary structure-based phylogenetic relationships among the enzymes, along with knowledge of the fatty acid profile of the source organisms of these acyl-ACP TEs. Briefly, at least one TE was characterized from each of the ten subfamilies except for Subfamily C, whose members appear to be specific for oleoyl-ACP substrates. For subfamilies that contain acyl-ACP TEs originating from organisms without any known fatty acid data, or from organisms where acyl-ACP TEs were not previously characterized, we chose to investigate acyl-ACP TE sequences that are evolutionarily distant from each other within each subfamily. For example, within Subfamily A there are two distinct and separate groupings of acyl-ACP TEs that are derived from the Poaceae family, for which there is no functional characterization (Table 1, containing refs. [29-35], and Additional file 2, Figure A1). One grouping contains one sorghum acyl-ACP TE sequence (GenBank:EER87824) and the other contains two (GenBank:EER88593 and GenBank:EES04698). To explore this structural divergence as an indicator of potential functional divergence in substrate specificities, one each of these Subfamily A sorghum acyl-ACP TEs (GenBank:EER87824 and GenBank:EER88593) and the two Subfamily B sorghum acyl-ACP TEs were expressed and functionally characterized.

**Table 1:** Total fatty acid production of synthesized and cloned acyl-ACP TEs

Kingdom	Subfamily	ACC No./Name	Organism	Rationale for synthesis*^a^*	Total FA*^b^* (nmol/mL)
Planta	A	AAC49179*^c, d^*	*Cuphea palustris*	A (Bimodal specificity for C8 and C10 substrates) [1]	708 ± 45

		AAB71731	*Ulmus americana*	A (Broad specificity; highest activity on C10 and C16) [13]	1098 ± 62

		AAG43857	*Iris germanica*	B	261 ± 20

		AAG43858	*Iris germanica*	B	14.8 ± 4.6

		EER87824	*Sorghum bicolor*	B (Member of a Subfamily A Poeceae TE cluster)	126 ± 13

		EER88593	*Sorghum bicolor*	B (Member of a Subfamily A Poeceae TE cluster)	90.7 ± 8.0

		CnFatB1	*Cocos nucifera*	C	130 ± 12

		CnFatB2	*Cocos nucifera*	C	572 ± 32

		CnFatB3	*Cocos nucifera*	C	200 ± 11

		CvFatB1	*Cuphea viscosissima*	C	79.2 ± 9.7

		CvFatB2	*Cuphea viscosissima*	C	249 ± 9

		CvFatB3	*Cuphea viscosissima*	C	18.9 ± 2.1

		AAD42220	*Elaeis guineensis*	C	36.7 ± 3.8

	B	EDQ65090	*Physcomitrella patens*	B (Member of novel plant subfamily)	380 ± 29

		EER96252	*Sorghum bicolor*	B (Member of novel plant subfamily)	175 ± 11

		EES11622	*Sorghum bicolor*	B (Member of novel plant subfamily)	9.43 ± 2.03

	D	EEH52851	*Micromonas pusilla*	B	16.3 ± 1.6

Bacteria	E	ACL08376	*Desulfovibrio vulgaris*	D (Medium-chain linear, branched, and hydroxy fatty acids) [29]	330 ± 9

	F	CAH09236	*Bacteroides fragilis*	D (Hydroxy fatty acids) [29]	215 ± 6

		ABR43801	*Parabacteroides distasonis*	D (Branched and branched hydroxy fatty acids) [30]	70.3 ± 4.4

		AAO77182*^e^*	*Bacteroides thetaiotao-*	D (Anteiso-branched and hydroxy fatty acids) [29]	60.4 ± 2.9

			*micron*		

	G	ABG82470	*Clostridium perfringens*	D (Medium-chain fatty acids) [31]	72.0 ± 9.5

	H	EEG55387	*Clostridium asparagiforme*	B	25.9 ± 4.2

		EET61113	*Bryantella formatexigens*	B	381 ± 3

	I	EDV77528	*Geobacillus sp*.	D (Iso-branched fatty acids) [32]	64.9 ± 12.0

	J	BAH81730	*Streptococcus dysgalactiae*	D (Medium-chain and cyclic propane ring fatty acids) [29]	623 ± 14

		ABJ63754	*Lactobacillus brevis*	D (Medium-chain and cyclic propane ring fatty acids) [33]	710 ± 10

		CAD63310*^e^*	*Lactobacillus plantarum*	D (Medium-chain 3'-hydroxy fatty acids) [33,34]	436 ± 10

	Non-grouped	EEI82564	*Anaerococcus tetradius*	D (Organism produces butyric acid) [35]	1381 ± 146

		CAE80300	*Bdellovibrio bacteriovorus*	D (Straight-chain odd-numbered fatty acids) [29]	333 ± 18

		ABN54268	*Clostridium thermocellum*	D (Branched-chain fatty acids) [29]	97.7 ± 3.2

*^a ^*A: Functionally characterized TEs; B: TE does not group near characterized TEs and/or no organism lipid profile information is available; C: TEs cloned from organisms known to produce MCFAs; D: Organism's lipid profile used and predominant fatty acid constituents identified in the organism are listed in parentheses.

*^b ^*The data are represented as mean ± standard error (*n *= 4).

*^c ^*All but the three *C. nucifera *sequences were codon-optimized for expression in *E. coli*.

*^d ^*Transit peptides were removed from all plant sequences.

*^e ^*Acyl-ACP TEs with known crystal structures.

TEs were expressed in *E. coli *K27, and free fatty acids (FAs) that accumulated in the medium were analyzed by GC-MS.

### Isolation and sequence analysis of acyl-ACP TEs from coconut and *C. viscosissima*

MCFAs are abundant in the oil produced in fruits of coconut (i.e. predominantly C12 and C14 and a small amount (0.2-1%) of C6 fatty acids [36-38]) and seeds of *C. viscosissima *(i.e. predominantly C8 and C10 fatty acids [39]). Therefore, acyl-ACP TEs in the seeds of these species are predicted to be specific for medium-chain acyl-ACPs. Acyl-ACP TE sequences were isolated from coconut and *C. viscosissima *by a homologous cloning strategy. Using degenerate primers, which were designed from conserved regions of plant TE14 family enzymes, a 350-bp fragment in the middle of the mRNAs was amplified from cDNA generated from both developing coconut endosperm and *C. viscosissima *seeds. Sequencing of cloned PCR products identified three new acyl-ACP TE sequences each from coconut and *C. viscosissima*. The full-length cDNA sequences were obtained by RACE for three acyl-ACP TEs [CnFatB1 (JF338903), CnFatB2 (JF338904), and CnFatB3 (JF338905)] from coconut and three [CvFatB1 (JF338906), CvFatB2 (JF338907), and CvFatB3 (JF338908)] from *C. viscosissima*.

**Figure 3:**
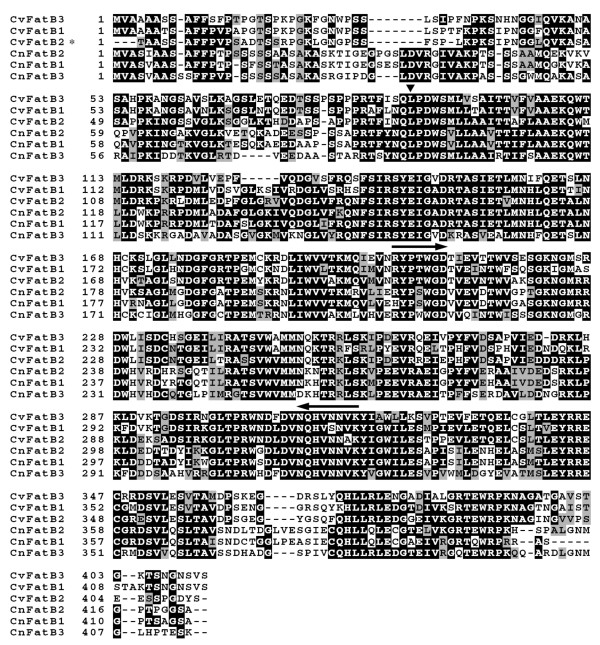
**Sequence alignment of deduced amino acid sequences of *C. nucifera *(Cn) and *C. viscosissima *(Cv) acyl-ACP TEs**. The putative N-terminal amino acid residue is leucine (▼). Two arrows indicate the conserved regions from which the degenerated primers were designed. The N-terminal sequence of CvFatB2 is incomplete (*).

The predicted open reading frames of coconut and *C. viscosissima *acyl-ACP TE cDNAs were identified. They encode pre-proteins of 412 to 423 amino acids, with calculated molecular weights of 45.8 to 46.5 kDa and theoretical pIs of 6.4 to 8.8. Plant acyl-ACP TEs are nuclear-encoded, plastid-targeted proteins with an N-terminal plastid-targeting peptide extension [2]. For each of the cloned coconut and *C. viscosissima *acyl-ACPs TEs, the putative plastid-targeting peptide cleavage site was located on the N-terminal side of the conserved sequence LPDW (Figure 3), as proposed for many other plant acyl-ACP TEs [5,8,12,40,41]. These yield predicted mature proteins of 323 to 331 amino acid residues [42], with calculated molecular weights of 36.6 to 37.5 kDa and theoretical pIs of 5.4 to 7.3. Alignment of the deduced amino acid sequences of coconut and *C. viscosissima *acyl-ACP TE cDNAs showed that, except for the plastid-targeting peptide sequences and very near the C-terminus, the sequences are colinear and share very high identity (63-86%) within a species (Figure 3). These sequences cluster within Subfamily A (Additional file 2, Figure A1).

### Determination of *in vivo *activities of acyl-ACP TEs

All isolated acyl-ACP TE cDNAs were expressed in *E. coli *strain K27. Secreted fatty acids were analyzed with GC-MS, and the total fatty acid yield in the medium was used to represent the *in vivo *activities of these enzymes on acyl-ACPs, though it remains possible that some of these enzymes might also hydrolyze acyl-CoAs [43].

**Figure 4:**
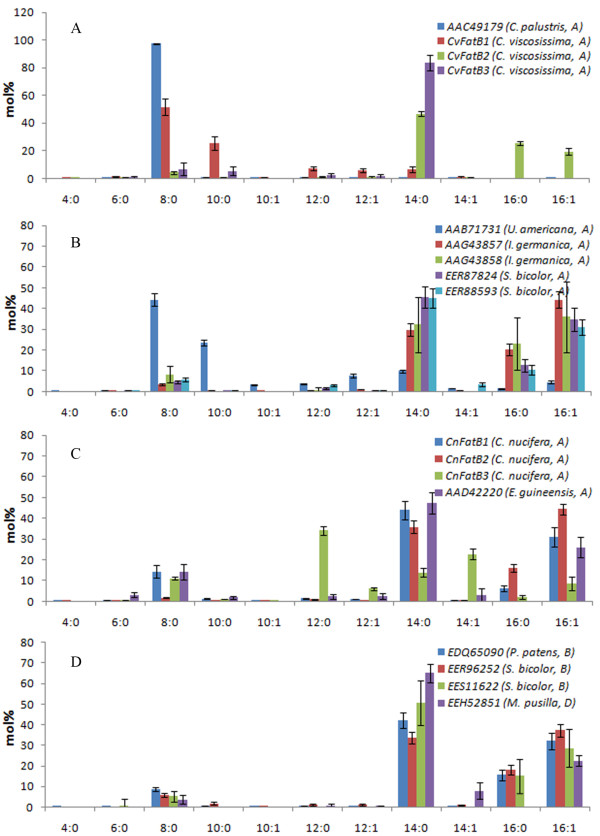
**Fatty acid compositions of *E. coli *K27 cultures expressing plant acyl-ACP TEs**. A: TEs from coconut and oil palm in Subfamily A; B: TEs from *C. viscosissima *and *Cuphea palustris *in Subfamily A; C: remaining TEs characterized from Subfamily A; D: TEs in Subfamily B and Subfamily D. In parentheses are the organism and subfamily from which each sequence belongs. Error bars represent standard errors.

A total of 13 acyl-ACP TEs from Subfamily A were characterized, including single acyl- ACP TEs from *Cuphea palustris *(GenBank:AAC49179)*, U. americana *(GenBank:AAB71731), and oil palm (*E. guineensis*, GenBank:AAD42220), two each from *Iris germanica *(GenBank:AAG43857 and GenBank:AAG43858) and *Sorghum bicolor *(GenBank:EER87824 and GenBank:EER88593), and three each from coconut and *C. viscosissima*. Total fatty acid concentrations produced by these acyl-ACP TEs are listed in Table 1, and the resulting fatty acid compositions are shown in Figure 4 and Additional file 12, Table A2. Acyl-ACP TEs from *C. palustris *and *U. americana*, which have previously been functionally characterized *in vitro *[1,13], were studied as controls.

*C. palustris *acyl-ACP TE produced 97 mol% 8:0 and only 0.8 mol% 10:0 fatty acids (Figure 4A), while *U. americana *acyl-ACP TE made 44 mol% 8:0 and 23 mol% 10:0 fatty acids (Figure 4B). *E. guineensis *acyl-ACP TE produced mainly 14:0 (47 mol%) and 16:1 (26 mol%) fatty acids (Figure 4C). The acyl-ACP TEs from *I. germanica *and *S. bicolor *have similar substrate specificities, producing mainly 14:0 (30-46 mol%), 16:0 (11-23 mol%), and 16:1 (31-44 mol%) fatty acids (Figure 4B). CnFatB1 (JF338903) and CnFatB2 (JF338904) made predominantly 14:0 (36-44 mol%) and 16:1 (31-44 mol%) fatty acids, whereas CnFatB3 (JF338905) made mainly 12:0 (34 mol%) and 14:1 (22 mol%) fatty acids (Figure 4C). Finally, CvFatB1 (JF338906) produced mainly 8:0 (51 mol%) and 10:0 (25 mol%), and CvFatB2 (JF338907) made mainly 14:0 (46 mol%), 16:0 (25 mol%) and 16:1 (20 mol%) fatty acids (Figure 4A). In contrast, CvFatB3 (JF338908) has narrower substrate specificity, producing predominantly 14:0 fatty acid (84 mol%).

Three acyl-ACP TEs from plant sources belonging to Subfamily B, including those from *P. patens *(GenBank:EDQ65090) and *S. bicolor *(GenBank:EER96252 and GenBank:EES11622), and one acyl-ACP TE from Subfamily D sourced from the alga *Micromonas pusilla *(GenBank:EEH52851), were similarly characterized. Total fatty acid production in *E. coli *expressing these acyl-ACP TEs varied from 9 to 380 nmol/mL (Table 1). These four acyl-ACP TEs showed similar substrate specificities, producing predominantly 14:0 (34-65 mol%) and 16:1 (23-37 mol%) fatty acids (Figure 4D).

**Figure 5:**
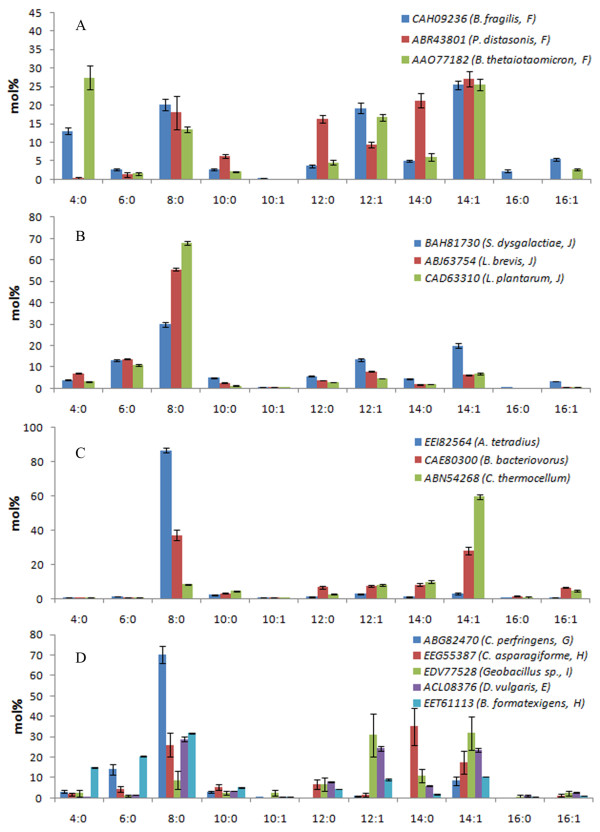
**Fatty acid compositions of *E. coli *K27 cultures expressing bacterial acyl-ACP TEs**. A: TEs from Subfamily F; B: TEs from Subfamily J; C: non-grouped TEs; D: other bacterial TEs. In parentheses are the organism, and for A, B and D, the subfamily from which each sequence belongs (non-grouped sequences are found in C). Error bars represent standard errors.

**Figure 6:**
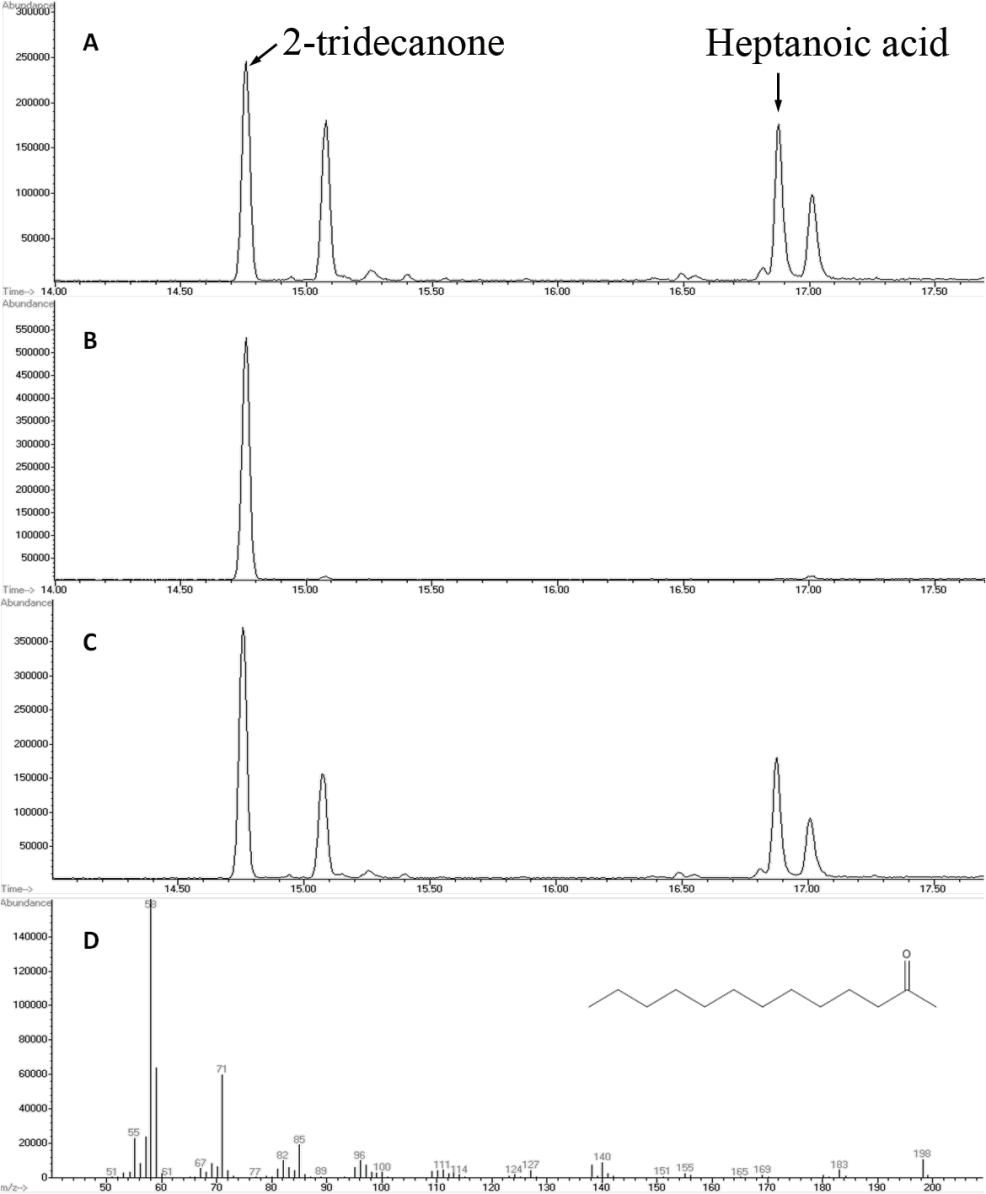
**Identification of 2-tridecanone in the culture expressing a bacterial TE**. A: GC of extract from *E. coli *K27 culture expressing a bacterial TE (*Bdellovibrio bacteriovorus*, GenBank:CAE80300); B: GC of 2-tridecanone standard; C: GC of a mixture of A and B; D: mass spectrum of 2-tridecanone.

Eleven acyl-ACP TE sequences from Subfamilies E to J sourced from bacteria and three bacterial sequences not placed in any subfamily were characterized (Table 1, Figure 5, and Additional file 12, Table A2). Based on their substrate specificities, these acyl-ACP TEs were classified into two groups. One group produced primarily SCFAs and MCFAs (> 75 mol% 4:0 to 8:0 fatty acids). This group included acyl-ACP TEs from *Anaerococcus tetradius *(GenBank:EEI82564, no subfamily, 87% 8:0), *Clostridium perfringens *(GenBank:ABG82470, Subfamily G, 14% 6:0 and 70% 8:0), *Lactobacillus brevis *(GenBank:ABJ63754, Subfamily J, 7% 4:0, 14% 6:0, and 55% 8:0), and *Lactobacillus plantarum *(GenBank:CAD63310, Subfamily J, 11% 6:0 and 68% 8:0) (Figure 5 and Additional file 12, Table A2). The other group showed broad- and binary-range substrate specificities. The binary-range activities were centered on C8 and C12/C14 substrates (Figure 5). Interestingly, many bacterial acyl-ACP TEs, such as those from *Desulfovibrio vulgaris *(GenBank:ACL08376, Subfamily E), *L. brevis *(GenBank:ABJ63754, Subfamily J), *L. plantarum *(GenBank:CAD63310, Subfamily J), and *Bdellovibrio bacteriovorus *(GenBank:CAE80300, no subfamily), are part of the pathway that produces noticeable amounts of the methylketone 2-tridecanone through enzymatic hydrolysis of 3-keto-tetradecanoyl-ACP followed by chemical decarboxylation (data not shown). *B. bacteriovorus *acyl-ACP TE produced the highest concentration of 2-tridecanone, 9.4 nmol/mL (Figure 6), which was 3 mol% of the fatty acids produced.

### Clustering acyl-ACP TEs based on their catalytic functionality

**Figure 7:**
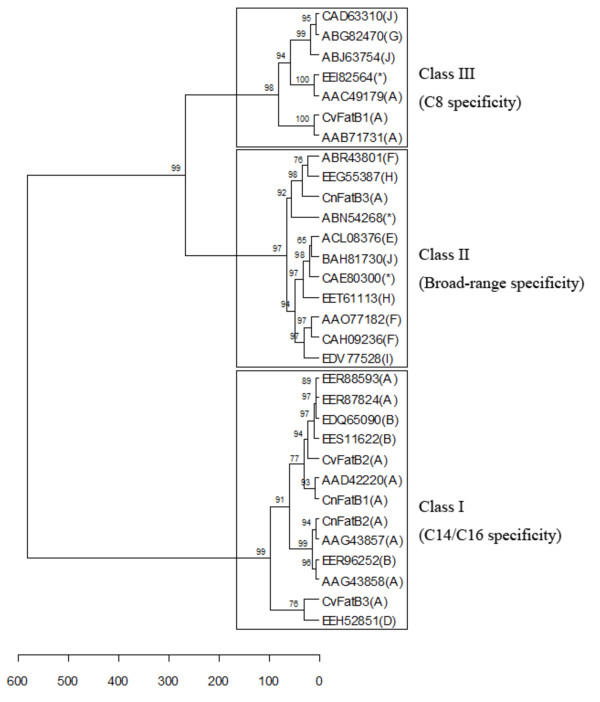
**Hierarchical clustering dendrogram of acyl-ACP TEs**. Cluster analysis was performed with fatty acid composition data using Euclidean distances and Ward's hierarchical clustering method. The *p*-values were calculated via multiscale bootstrap resampling with 1000 replicates. Subfamilies to which each sequence belongs are indicated in parentheses. Non-grouped sequences are indicated by asterisks.

To classify acyl-ACP TEs based on their substrate specificities, cluster analysis was performed on the fatty acid composition data as described in the Experimental Procedures. All acyl-ACP TEs characterized in this study clustered into three classes: 1) Class I contains acyl-ACP TEs that mainly act on C14 and C16 substrates; 2) Class II has acyl-ACP TEs that have broad substrate specificities, with major activities toward C8 and C14 substrates; and 3) Class III comprises acyl-ACP TEs that predominantly act on C8 substrate (Figure 7). Class I consists of thirteen plant acyl-ACP TEs from Subfamilies A, B, and D. Class II contains eleven acyl-ACP TEs, ten from bacteria in Subfamilies E, F, H, I, and J, and a non-grouped sequence, and only one from a plant (CnFatB3) in Subfamily A. Class III includes seven acyl-ACP TEs, of which three are from plants in Subfamily A and four are from bacteria in Subfamilies G and J and a non-grouped sequence. Considering the previously characterized class of oleoyl-ACP TEs in Subfamily C, TE14 members may now be sorted into four classes based on their substrate specificities.

## Discussion

### The systematic functional characterization of bacterial acyl-ACP TEs demonstrates production of SCFAs

Over the past few decades, the number of acyl-ACP TE sequences in public databases has increased exponentially. The vast majority of these annotations are based solely on primary sequence homology; most have not been functionally characterized. The difficulty of purifying protein and preparing substrates precludes a large-scale *in vitro *characterization of acyl-ACP TEs. However, the well-known and widely used approach of analyzing fatty acid concentrations and distributions produced by heterologous TEs expressed in *E. coli *K27 provided an efficient and fast way to study the activities of a large number of diverse acyl-ACP TEs. The integration of phylogeny and prior knowledge of the fatty acid profiles of the source organisms for these enzymes allowed us to rationally choose a representative subset of 31 acyl-ACP TEs to characterize. Significantly, this study represents the first experimental validation and functional characterization of bacterial acyl-ACP TEs, 14 of which were studied here.

Seven of these bacterial acyl-ACP TEs, those from *Bacteroides fragilis *(GenBank:CAH09236, Subfamily F), *B. thetaiotaomicron *(GenBank:AAO77182, Subfamily F), *Clostridium asparagiforme *(GenBank:EEG55387, Subfamily H), *Bryantella formatexigen *(GenBank:EET61113, Subfamily H), *L. brevis *(GenBank:ABJ63754, Subfamily J), *L. plantarum *(GenBank:CAD63310, Subfamily J), and *Streptococcus dysgalactiae *(GenBank:BAH81730, Subfamily J) produced significant amounts of 4:0 and 6:0 fatty acids when expressed in *E. coli*. This is the first report of acyl-ACP TEs that have these catalytic activities. Although these enzymes did not appear to show high activities against C4-ACP and C6-ACP, they provide a good starting point for protein engineering. Both of these SCFAs could then be potential candidates for platform biochemicals for a biorenewable chemical industry [4].

### Acyl-ACP TEs from MCFA-producing plant tissues make MCFAs

Plant tissues known to produce MCFAs were shown here to contain at least one acyl-ACP TE specific for medium chain acyl-ACPs. It appears that CnFatB1 (producing primarily 8:0, 14:0, and 16:0 fatty acids), CnFatB2 (making mainly 14:0, 16:0, and 16:1 fatty acids) and CnFatB3 (producing mostly 8:0, 12:0, 14:0, and 14:1 fatty acids) might work together to determine the fatty acid composition of coconut oil, which contains primarily 12:0 (43-50%) and 14:0 (16-22%) and small amounts of 6:0, 8:0, and 10:0 fatty acids [36-38]. However, we cannot rule out the possibility that other acyl-ACP TEs are also expressed in coconut endosperm, and that they may be involved in determining the fatty acid composition of the oil.

The CvFatB1 and CvFatB3 TEs, for which corresponding cDNAs were isolated from the developing seeds of *C. viscosissima *produced MCFAs in *E. coli*, and CvFatB1 shows substrate specificity consistent with the fatty acid constituents present in the seed oil. The relative distributions of 8:0 and 10:0 fatty acids differ; CvFatB1 produced twice as much 8:0 compared to 10:0 fatty acid, whereas there is ~fourfold more 10:0 fatty acid within *C. viscosissima *seed oil [39]. Differences in *in vivo *substrate activities in *E. coli *K27 compared to *in vitro *enzymatic assays, or in the fatty acid composition of the organism from which the acyl-ACP TE was sourced, have been noted previously [1,10,11,13], and could possibly apply to non-plant TEs as well. This phenomenon may reflect the complexity of the fatty acid biosynthesis pathway within the plant. For example, multiple acyl-ACP TEs within an organism may contribute to fatty acid composition. Alternatively, the fatty acid profile of an organism may be determined by the kinetics of the entire fatty acid biosynthesis pathway, as has been previously proposed [11,25,44], including the contribution made by the species-specific interactions between the acyl-ACP TE and the ACP molecule that carries the acyl-substrate for the acyl-ACP TE. Regardless, this study identifies specific medium-chain substrates on which the TE can act, which is especially important for engineering the fatty acid biosynthesis pathway.

### Acyl-ACP TEs can intercept both saturated and unsaturated intermediates of Type II fatty acid synthase of *E. coli*

Several plant acyl-ACP TEs (e.g. CnFatB3) produced significant amounts of unsaturated fatty acids (UFAs) when expressed in *E. coli*. These include 10:1, 12:1, 14:1, and 16:1 fatty acids (Figure 4), which do not usually accumulate in *E. coli *or in the original host plant tissues from which the acyl-ACP TE was isolated. A similar finding has been reported for a *U. californica *acyl-ACP TE expressed in *E. coli *K27 [25]. Although the double bond position within these fatty acids was not determined in this study, double bonds in UFAs produced in *E. coli *K27 expressing a *Cinnamonum camphorum *acyl-ACP TE were all in *cis *conformation and at the ω - 7 position [45]. *E. coli *has a different UFA biosynthesis pathway than plants. Bacteria, such as *E. coli*, utilize a different anaerobic system in which the double bond is retained in the acyl chain as it is being assembled. Plants instead use aerobic acyl-ACP desaturase to introduce double bonds into the acyl chain once it is preformed. Specifically, the FabA gene in *E. coli*, encoding 3-hydroxydecanoyl-ACP dehydratase/isomerase, is a bifunctional enzyme that introduces a double bond at C10 and regulates the branch point of the saturated and unsaturated pathways [46]. FabB encodes a 3-ketoacyl-ACP synthase that catalyzes the elongation of *cis*-3-decenoyl-ACP produced by FabA [46]. Because 10:1-ACP, 12:1-ACP, 14:1-ACP, and 16:1-ACP are intermediates of the UFA biosynthesis pathway in *E. coli*, the UFAs produced by acyl-ACP TEs are most likely derived from those intermediates and thus are in the *cis *conformation and unsaturated at the ω - 7 position. The accumulation of both UFAs and saturated fatty acids observed in this study is consistent with the previous conclusion that the heterologously expressed acyl-ACP TEs can intercept both saturated and unsaturated intermediates of fatty acid biosynthesis of *E. coli *[46].

### Subtle changes in primary sequences may be sufficient to change the substrate specificity of acyl-ACP TEs

The relationship between the structures of acyl-ACP TEs and their functionalities (i.e. their substrate specificities) is poorly understood. To begin to address this question, the 31 acyl-ACP TEs that were functionally characterized herein were clustered using the substrate specificity data obtained from their *in vivo *activities (Figures 4 and 5 and Additional file 12, Table A2). Comparison between the specificity-based classification and the sequence-based phylogenetic tree (Figure 1) indicates that the two classifications are not necessarily consistent with each other. Three phenomena were observed in this study. First, diverged sequences (variants in primary structure) from the same species do not necessarily differ in function. For example, *S. bicolor *expresses at least three acyl-ACP TEs in Subfamily A and two in Subfamily B, all of which share very similar substrate specificity as measured by the fatty acids produced when expressed in *E. coli *(Figure 4D). One possible explanation for the persistence of this number of acyl-ACP TEs with similar function within a species genome may be due to divergence in spatial or temporal expression of their acyl-ACP TEs. Second, similar sequences may have different substrate specificities, e.g., three acyl-ACP TEs from *C. viscosissima *have different substrate specificities although their mature protein sequences share more than 70% primary sequence identity, and they all are classified within Subfamily A. Third, sequences that belong to different subfamilies because they share low sequence identity can have very similar substrate specificities. For example, CnFatB2 (Subfamily A) and *S. bicolor *(GenBank:EER96252, Subfamily B) acyl-ACP TEs are members of different subfamilies and share only 40% sequence identity, and yet they have very similar substrate specificities. Therefore, it is not reasonable to infer the substrate specificity of one acyl-ACP TE based on its sequence-based classification within the same subfamily. It is conceivable, therefore, that the change of substrate specificity is most likely caused by changes of only a few amino acid residues, and that many different combinations of residue changes could result in changed specificities [5]. In previous studies of FatA and FatB TEs, discrete sequence changes in a region of the putative ACP binding site [7] or in residues surrounding the catalytic site [47] altered substrate specificity. These studies were both based on predicted structures. Identifying the amino acids that determine substrate specificity is critical for engineering novel acyl-ACP TEs, but this is limited by the lack of tertiary structural information of acyl-ACP TEs from different subfamilies. A comparison of the two PDB structures known for bacterial acyl-ACP TEs, from *B. thetaiotaomicron *(PDB:2ESS, GenBank:AAO77182, Subfamily F) and *L. plantarum *(PDB:2OWN, GenBank:CAD63310, Subfamily J), is instructive. Although they share only 18% sequence identity, these two proteins share a common HotDog tertiary structure, being co-aligned with an RMSD of 2.59 Å (Figure 2). However, *B. thetaiotaomicron *acyl-ACP TE has broad substrate specificity, while *L. plantarum *acyl-ACP TE is specific for C6 and C8 acyl-ACP substrates. Thus, future work can focus on identifying and validating the role of specific residues in determining acyl-ACP TE substrate specificity.

### Unexpected activity reveals diversity of acyl-ACP TEs

Interestingly, in the *E. coli *heterologous expression system used here, six bacterial-sourced acyl-ACP TEs and three plant-sourced acyl-ACP TEs produced noticeable amounts (> 1 nmol/mL) of methylketones, largely 2-tridecanone. The acyl-ACP TE from *B. bacteriovorus *(GenBank:CAE80300) produced the highest concentration of 2-tridecanone (9.4 nmol/mL).

Methylketones such as 2-tridecanone occur in the wild tomato species *Solanum habrochaites *subsp. *Glabratum *[48], and their biosynthesis is catalyzed by two sequentially-acting methylketone synthases, MKS1 and MKS2. MKS2 is a TE that catalyzes the hydrolysis of the 3-ketoacyl-ACP intermediate in fatty acid biosynthesis, and MKS1 catalyzes the decarboxylation of the released 3-keto acid to produce a methylketone [49,50]. Heterologous expression of MKS2 in *E. coli *yields many methylketones, including 2-tridecanone [50]. However, MKS2 is not included in Family TE14, but instead it belongs to Family TE9 [14]. Although some Family TE14 members share very low if any significant sequence similarity (i.e., < 15% identity) to MKS2, the current study indicates that at least nine acyl-ACP TEs (e.g. *B. bacteriovorus*, GenBank:CAE80300) can catalyze the same reaction as MKS2 (i.e, hydrolysis of the thioester bond of 3-ketoacyl-ACP), and that the resulting product (3-keto acid) is further chemically or enzymatically decarboxylated to generate the methylketone. The ^β^-ketoacyl decarboxylase activity involved in methylketone production in both the fungus *Penicillium roqueforti *[51] and the bacterium *Staphylococcus carnosus *[52] has been described previously. Hence we cannot rule out the possibility that some ^β^-ketoacyl decarboxylase activity may also exist in *E. coli*.

## Conclusions

This study has revealed that acyl-ACP TEs isolated from different taxa have considerable functional diversity relative to their substrate specificity. Prior characterizations of plant acyl-ACP TEs have focused on the substrate specificity relative to acyl chain lengths, to identify such enzymes for bioengineering a source of lauric acid for use by the detergent industry. The present study has revealed that bacterial orthologs provide access to additional functional diversity, both relative to acyl chain length specificity (e.g., shorter acyl chains, as short as four carbon atoms), as well as acyl chains that contain additional chemical functionalities (e.g., unsaturated acyl chains and acyl chains containing carbonyl groups). This additional functional diversity in acyl-ACP TEs can potentially be used to diversify the fatty acid biosynthesis pathway to produce biorenewable chemicals [4].

## Authors' contributions

FJ and DCC contributed equally to the work. This body of work represents a collaboration between the Reilly and Nikolau laboratories. Graduate student DCC and undergraduate JPC conducted the computational and phylogenetic research in the Reilly laboratory. In the Nikolau laboratory graduate student FJ and Ames High School (Ames, IA) student JT performed the molecular and biochemical research with the aid and supervision of research scientist MDY-N. FJ, DCC, BJN, MDY-N, and PJR wrote the manuscript. All authors read and approved the final manuscript.

## Supplementary Material

Additional file 1 **Table A1: Mean JTT distances and *z*-values (bolded) within and between different subfamilies**. Click here for file

Additional file 2 **Figure A1: Rooted phylogenetic tree of Subfamily A**. Black diamonds mark genes that were synthesized for functional characterization, and black circles mark three coconut and three *Cuphea viscosissima *sequences isolated in this study. Click here for file

Additional file 3 **Figure A2: Rooted phylogenetic tree of Subfamily B**. Black diamonds mark genes that were synthesized for functional characterization. Click here for file

Additional file 4 **Figure A3: Rooted phylogenetic tree of Subfamily C**. Click here for file

Additional file 5 **Figure A4: Rooted phylogenetic tree of Subfamily D**. Black diamonds mark genes that were synthesized for functional characterization. Click here for file

Additional file 6 **Figure A5: Rooted phylogenetic tree of Subfamily E**. Black diamonds mark genes that were synthesized for functional characterization. Click here for file

Additional file 7 **Figure A6: Rooted phylogenetic tree of Subfamily F**. Black diamonds mark genes that were synthesized for functional characterization, and the black square marks a sequence with a known PDB structure. Click here for file

Additional file 8 **Figure A7: Rooted phylogenetic tree of Subfamily G**. Black diamonds mark genes that were synthesized for functional characterization. Click here for file

Additional file 9 **Figure A8: Rooted phylogenetic tree of Subfamily H**. Black diamonds mark genes that were synthesized for functional characterization. Click here for file

Additional file 10 **Figure A9: Rooted phylogenetic tree of Subfamily I**. Black diamonds mark genes that were synthesized for functional characterization. Click here for file

Additional file 11 **Figure A10: Rooted phylogenetic tree of Subfamily J**. Black diamonds mark genes that were synthesized for functional characterization. Click here for file

Additional file 12 **Table A2: Molar percentages and total concentrations of fatty acids produced by different TEs**. Click here for file
