# Supplementary material for: Phylogenetic and experimental characterization of an acyl-ACP thioesterase family reveals significant diversity in enzymatic specificity and activity
Source: BMC Biochem. 2011 Aug 10;12:44. doi: 10.1186/1471-2091-12-44 (PMC3176148; doi:10.1186/1471-2091-12-44)
Supplement: Additional file 1 — Table A1: Mean JTT distances and z-values (bolded) within and between different subfamilies. [file 1471-2091-12-44-S1.DOCX]

Additional file 1, Table A1. Mean JTT distances and *z*-values (bolded) within and between different subfamilies

_________________________________________________________________________________________________________________

A B C D E F G H I J

_________________________________________________________________________________________________________________

A 0.36 ± 0.13*^a^*

—

B 0.68 ± 0.13 0.58 ± 0.20

**4.37** —

C 0.94 ± 0.10 0.97 ± 0.13 0.25 ± 0.10

**25.37** **10.95** —

D 1.40 ± 0.08 1.42 ± 0.11 1.29 ± 0.14 0.95 ± 0.30

**6.14 5.03 5.55** —

E 1.80 ± 0.17 1.85 ± 0.19 1.76 ± 0.20 1.77 ± 0.18 1.53 ± 0.37

**9.28 7.67 9.10 3.41** —

F 2.09 ± 0.12 2.19 ± 0.20 2.00 ± 0.17 2.04 ± 0.16 1.98 ± 0.20 0.46 ± 0.36

**22.37 18.53 20.95 9.26 8.20** —

G 2.04 ± 0.19 2.08 ± 0.19 2.01 ± 0.17 2.13 ± 0.18 2.02 ± 0.25 2.05 ± 0.23 1.13 ± 0.40

**17.24 13.96 17.18 7.57 5.76 11.80** —

H 2.12 ± 0.13 2.23 ± 0.18 2.08 ± 0.14 2.12 ± 0.19 2.05 ± 0.24 2.03 ± 0.22 1.81 ± 0.26 0.96 ± 0.24

**29.34 21.29 28.10 8.71 7.58 14.37 8.43** —

I 2.24 ± 0.16 2.32 ± 0.17 2.28 ± 0.19 2.37 ± 0.22 2.28 ± 0.26 2.37 ± 0.23 2.22 ± 0.26 2.38 ± 0.26 1.95 ± 0.34

**8.78 7.98 9.37 5.08 3.41 7.94 4.63 6.81** —

J 2.21 ± 0.13 2.24 ± 0.15 2.23 ± 0.15 2.36 ± 0.20 2.20 ± 0.22 2.16 ± 0.17 1.91 ± 0.17 2.19 ± 0.25 2.26 ± 0.21 1.23 ± 0.40

**29.37 20.72 29.69 9.73 8.05 15.06 8.47 15.98 5.10** —

_________________________________________________________________________________________________________________

*^a^* Standard deviation
