# Supplementary material for: Phylogenetic and experimental characterization of an acyl-ACP thioesterase family reveals significant diversity in enzymatic specificity and activity
Source: BMC Biochem. 2011 Aug 10;12:44. doi: 10.1186/1471-2091-12-44 (PMC3176148; doi:10.1186/1471-2091-12-44)
Supplement: Additional file 2 — Figure A1: Rooted phylogenetic tree of Subfamily A. Black diamonds mark genes that were synthesized for functional characterization, and black circles mark three coconut and three Cuphea viscosissima sequences isolated in this study. [file 1471-2091-12-44-S2.PDF]

Additional file 2, Fig. A1. Subfamily A phylogenetic tree.

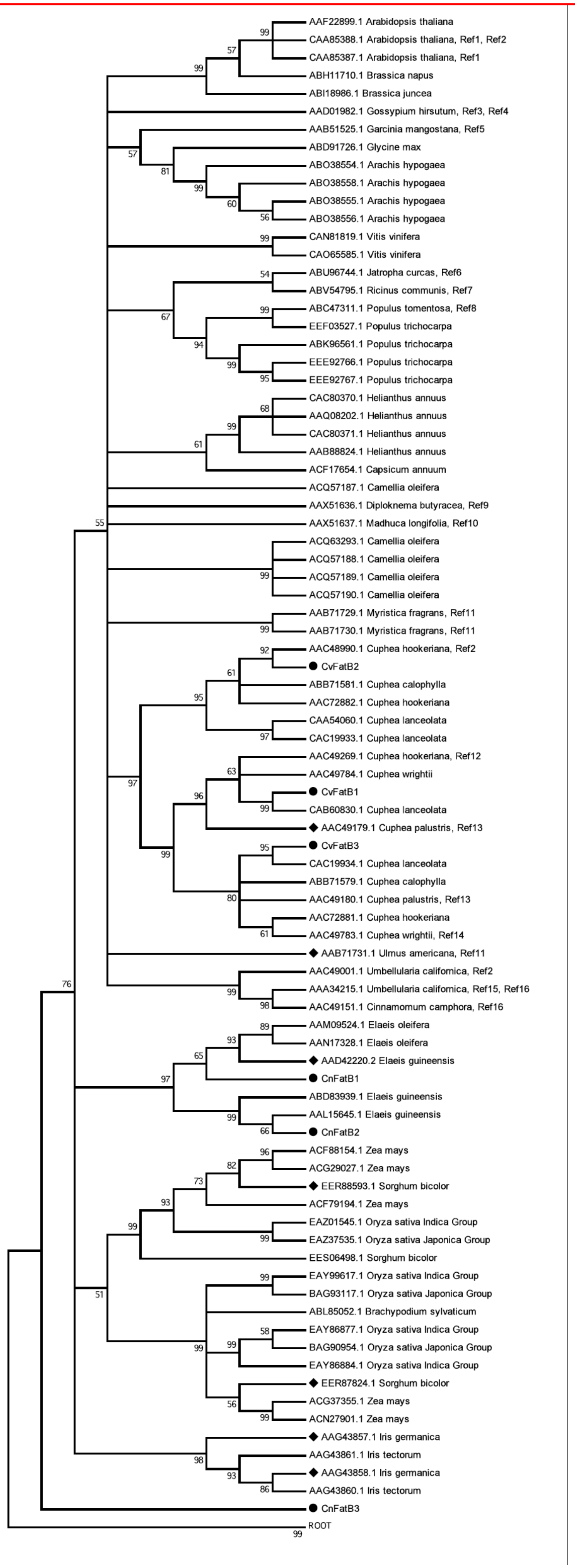

References to Citations Found in Figs. A1 and A3

1. Dormann P, Voelker TA, Ohlrogge JB: **Cloning and expression in *Escherichia coli* of a novel thioesterase from *Arabidopsis thaliana* specific for long-chain acyl-acyl carrier proteins.** *Arch Biochem Biophys* 1995, **316**:612–618.
2. Jones A, Davies HM, Voelker TA: **Palmitoyl-acyl carrier protein (ACP) thioesterase and the evolutionary origin of plant acyl-ACP thioesterases.** *Plant Cell 1995,* **7**:359–371.
3. Yoder DW, Nampaisansuk M, Pirtle IL, Chapman KD, Pirtle RM: **Molecular cloning and nucleotide sequence of a gene encoding a cotton palmitoyl-acyl carrier protein thioesterase.** *Biochim Biophys Acta 1999,* **1446**:403–413.
4. Pirtle RM, Yoder DW, Huynh TT, Nampaisansuk M, Pirtle IL, Chapman KD: **Characterization of a palmitoyl-acyl carrier protein thioesterase (FatB1) in cotton.** *Plant Cell Physiol 1999,* **40**:155–163.
5. Hawkins DJ, Kridl JC: **Characterization of acyl-ACP thioesterases from mangosteen (*Garcinia mangostana*) seed and high level of stearate**. *Plant J 1998,* **13**: 743–752.
6. Wu PZ, Li J, Wei Q, Zeng L, Chen YP, Li MR, Jiang HW, Wu GJ: **Cloning and functional characterization of an acyl-acyl carrier protein thioesterase (JcFatB1) from *Jatropha curcas*.** *Tree Physiol 2009,* **29**:1299–1305.
7. Sanchez-Garcia A, Moreno-Perez AJ, Muro-Pastor AM, Salas JJ, Garces R, Martinez-Force E: **Acyl-ACP thioesterase from castor (*Ricinus communis* L.): an enzymatic system appropiate for high rates of oil synthesis and accumulation.** *Phytochemistry 2010,* **71**:860–869.
8. Zhou Z, Zhang D, Lu M: **Cloning and expression analysis of PtFatB gene encoding the acyl-acyl carrier protein thioesterase in *Populus tomentosa* Carr.** *J Genet Genomics 2007,* **34**:267–273.
9. Jha JK, Maiti MK, Bhattacharjee A, Basu A, Sen PC, Sen SK: **Cloning and functional expression of an acyl-ACP thioesterase FatB type from *Diploknema* (*Madhuca*) *butyracea* seed in *Escherichia coli***. *Plant Physiol Biochem 2006,* **44**:645–655.
10. Ghosh SK, Bhattacharjee A, Jha JK, Mondal AK, Maiti MK, Basu A, Ghosh D, Ghosh S, Sen SK: **Characterization and cloning of a stearoyl/oleoyl specific fatty acyl-acyl carrier protein thioesterase from the seeds of *Madhuca longifoli* (*latifolia*).** *Plant Physiol Biochem 2007,* **45**:887–897.
11. Voelker TA, Jones A, Cranmer AM, Davies HM, Knutzon DS: **Broad-range and binary-range acyl-acyl carrier protein thioesterases suggest an alternative mechanism for medium-chain production in seeds.** *Plant Physiol 1997,* **114**:669–677.
12. Dehesh K, Jones A, Knutzon DS, Voelker TA: **Production of high levels of 8:0 and 10:0 fatty acids in transgenic canola by overexpression of Ch FatB2, a thioesterase cDNA from *Cuphea hookeriana***. *Plant J 1996,* **9**:167–172.
13. Dehesh K, Edwards P, Hayes T, Cranmer AM, Fillatti J: **Two novel thioesterases are key determinants of the bimodal distribution of acyl chain length of *Cuphea palustris* seed oil.** *Plant Physiol 1996,* **110**:203–210.
14. Leonard JM, Slabaugh MB, Knapp SJ: ***Cuphea wrightii* thioesterases have unexpected broad specificities on saturated fatty acids.** *Plant Mol Biol 1997,* **34**:669–679.
15. Voelker TA, Worrell AC, Anderson L, Bleibaum J, Fan C, Hawkins DJ, Radke SE, Davies HM: **Fatty acid biosynthesis redirected to medium chains in transgenic oilseed plants**. *Science 1992,* **25**:72–74.

16. Yuan L, Voelker TA, Hawkins DJ: **Modification of the substrate specificity of an acyl-acyl carrier protein thioesterase by protein engineering.** *Proc Natl Acad Sci USA 1995,* **92**:10639–10643

17. Loader NM, Woolner EM, Hellyer A, Slabas AR, Safford R: **Isolation and characterization of two *Brassica napus* embryo acyl-ACP thioesterase cDNA clones**. *Plant Mol Biol 1993,* **23**:769–778.

18. Knutzon DS, Bleibaum JL, Nelsen J, Kridl JC, Thompson GA: **Isolation and characterization of two safflower oleoyl-acyl carrier protein thioesterase cDNA clones**. *Plant Physiol 1992,* **100**:1751–1758.

19. Serrano-Vega MJ, Garces R, Martinez-Force E: **Cloning, characterization and structural model of a FatA-type thioesterase from sunflower seeds (*Helianthus annus* L.).** *Planta 2005,* **221**:868–880.

20. Dormann P, Kridl JC, Ohlrogge JB: **Cloning and expression in *Escherichia coli* of a cDNA coding for the oleoyl-acyl carrier protein thioesterase from coriander (*Coriandrum sativum* L.).** *Biochim Biophys Acta 1994,* **1212**:134–136.
