# Supplementary figures and images for: Phylogenetic and experimental characterization of an acyl-ACP thioesterase family reveals significant diversity in enzymatic specificity and activity
Source: BMC Biochem. 2011 Aug 10;12:44. doi: 10.1186/1471-2091-12-44 (PMC3176148; doi:10.1186/1471-2091-12-44)

Additional file 3, Fig. A2. Subfamily B phylogenetic tree.

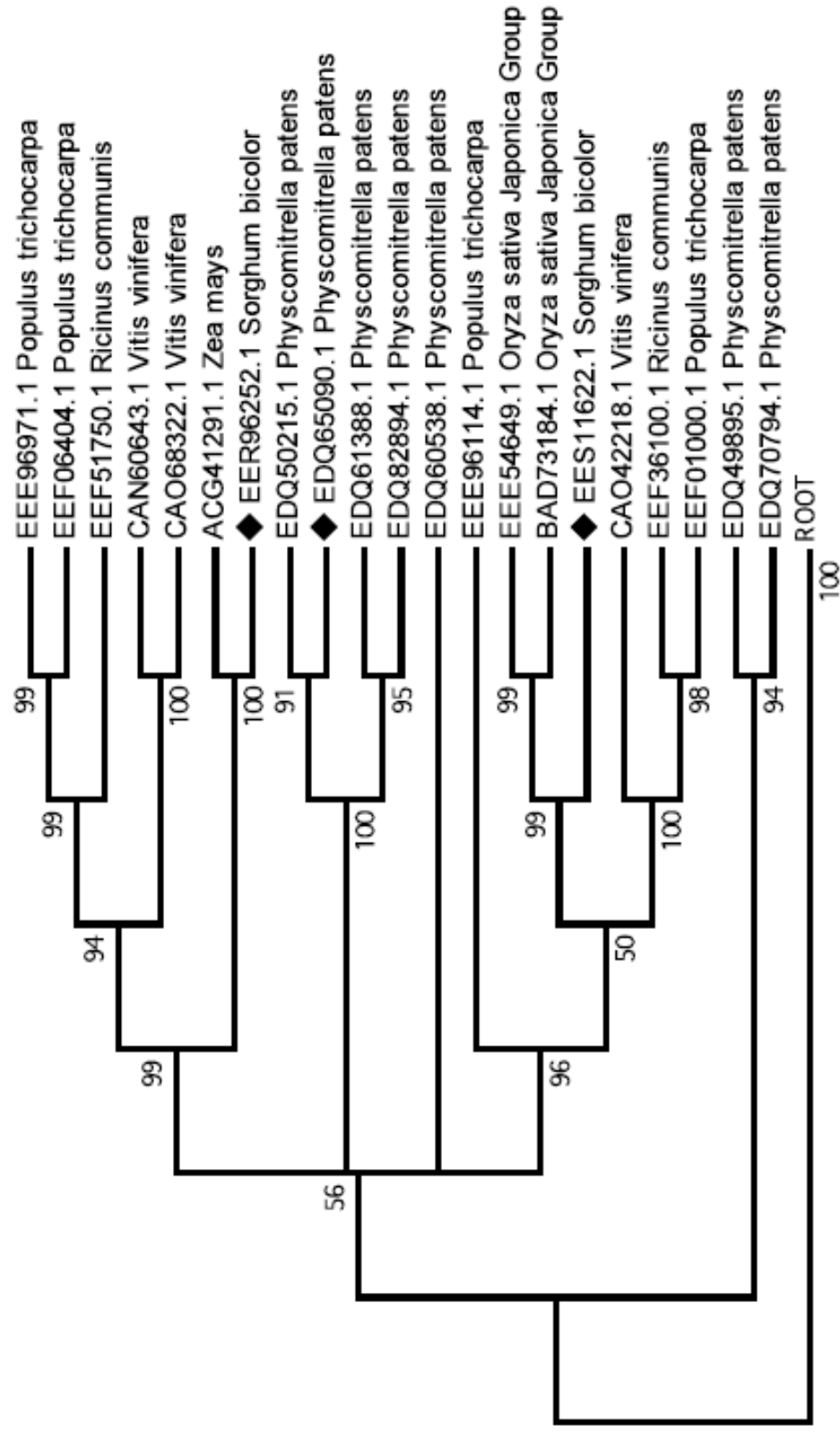

Supplement: Additional file 3 — Figure A2: Rooted phylogenetic tree of Subfamily B. Black diamonds mark genes that were synthesized for functional characterization. [file 1471-2091-12-44-S3.PDF]

Additional file 5, Fig. A4. Subfamily D phylogenetic tree.

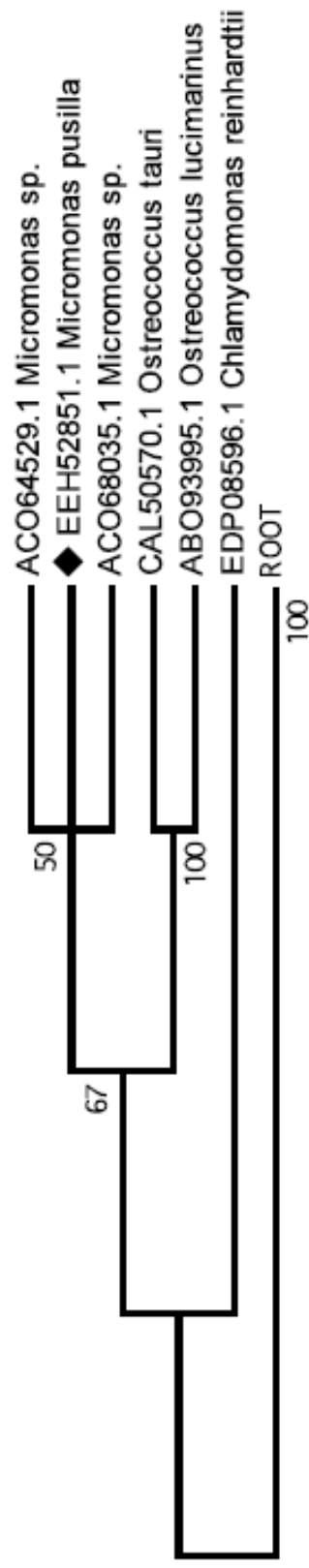

Supplement: Additional file 5 — Figure A4: Rooted phylogenetic tree of Subfamily D. Black diamonds mark genes that were synthesized for functional characterization. [file 1471-2091-12-44-S5.PDF]

Additional file 6, Fig. A5. Subfamily E phylogenetic tree.

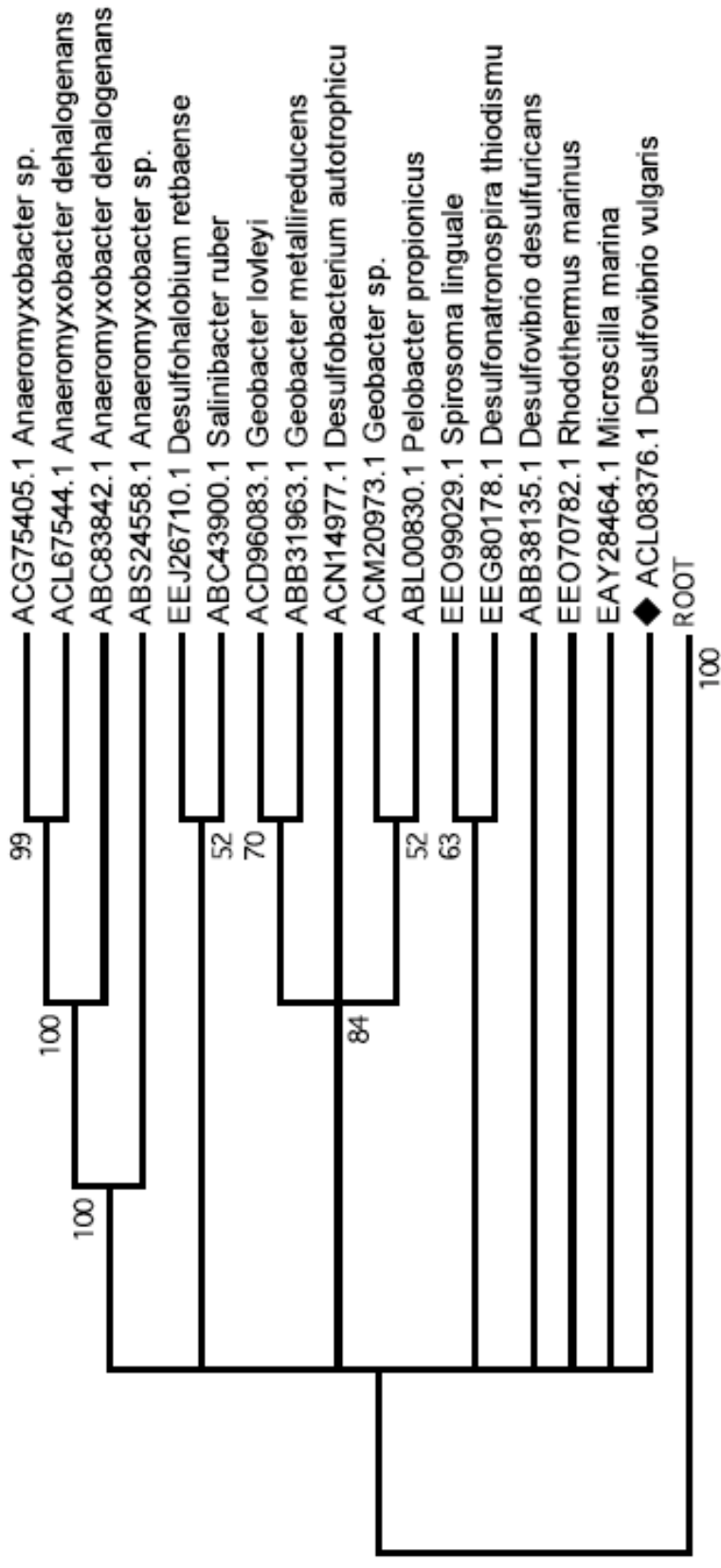

Supplement: Additional file 6 — Figure A5: Rooted phylogenetic tree of Subfamily E. Black diamonds mark genes that were synthesized for functional characterization. [file 1471-2091-12-44-S6.PDF]

Additional file 7, Fig. A6. Subfamily F phylogenetic tree.

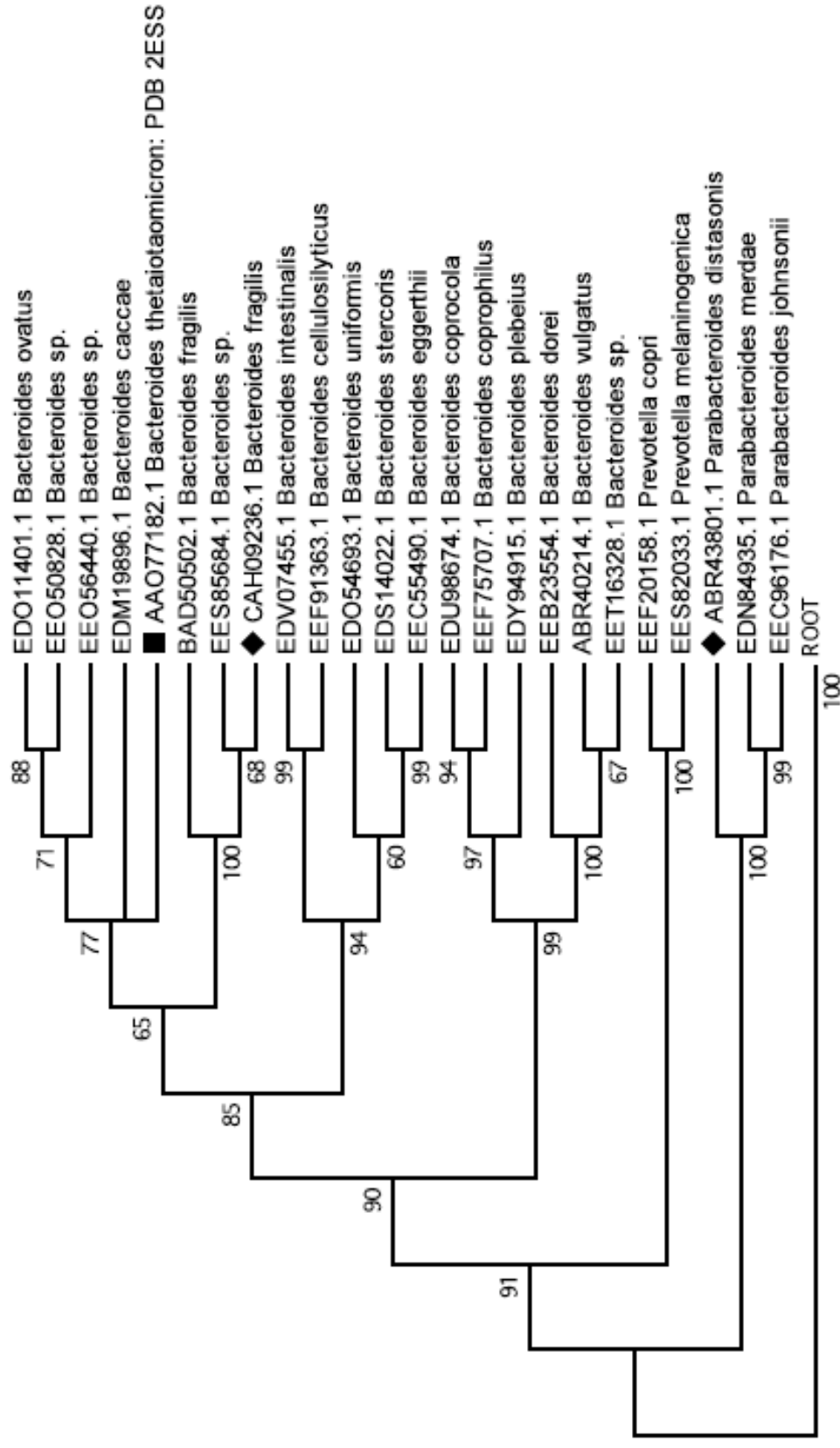

Supplement: Additional file 7 — Figure A6: Rooted phylogenetic tree of Subfamily F. Black diamonds mark genes that were synthesized for functional characterization, and the black square marks a sequence with a known PDB structure. [file 1471-2091-12-44-S7.PDF]

Additional file 8, Fig. A7. Subfamily G phylogenetic tree.

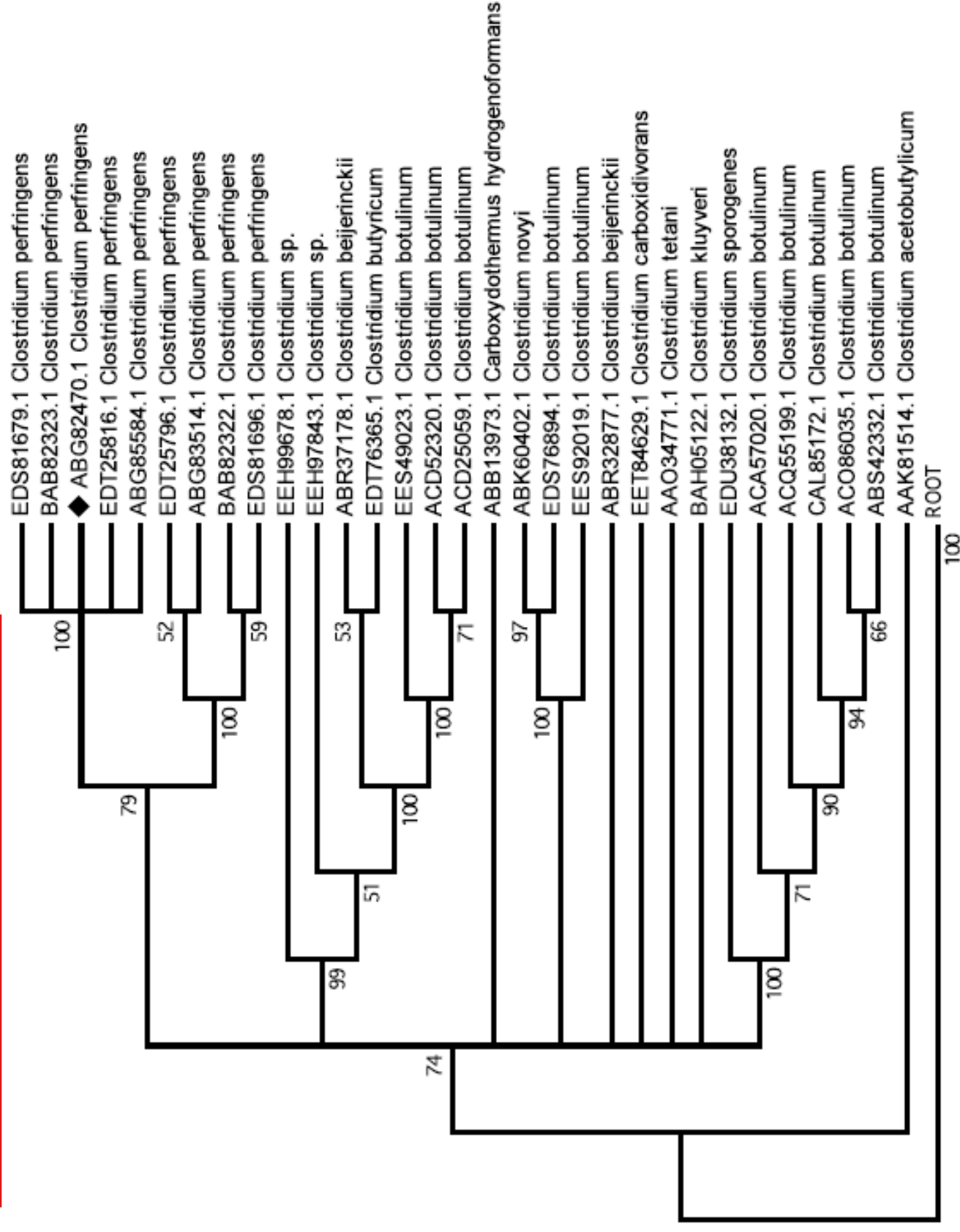

Supplement: Additional file 8 — Figure A7: Rooted phylogenetic tree of Subfamily G. Black diamonds mark genes that were synthesized for functional characterization. [file 1471-2091-12-44-S8.PDF]

Additional file 9, Fig. A8. Subfamily H phylogenetic tree.

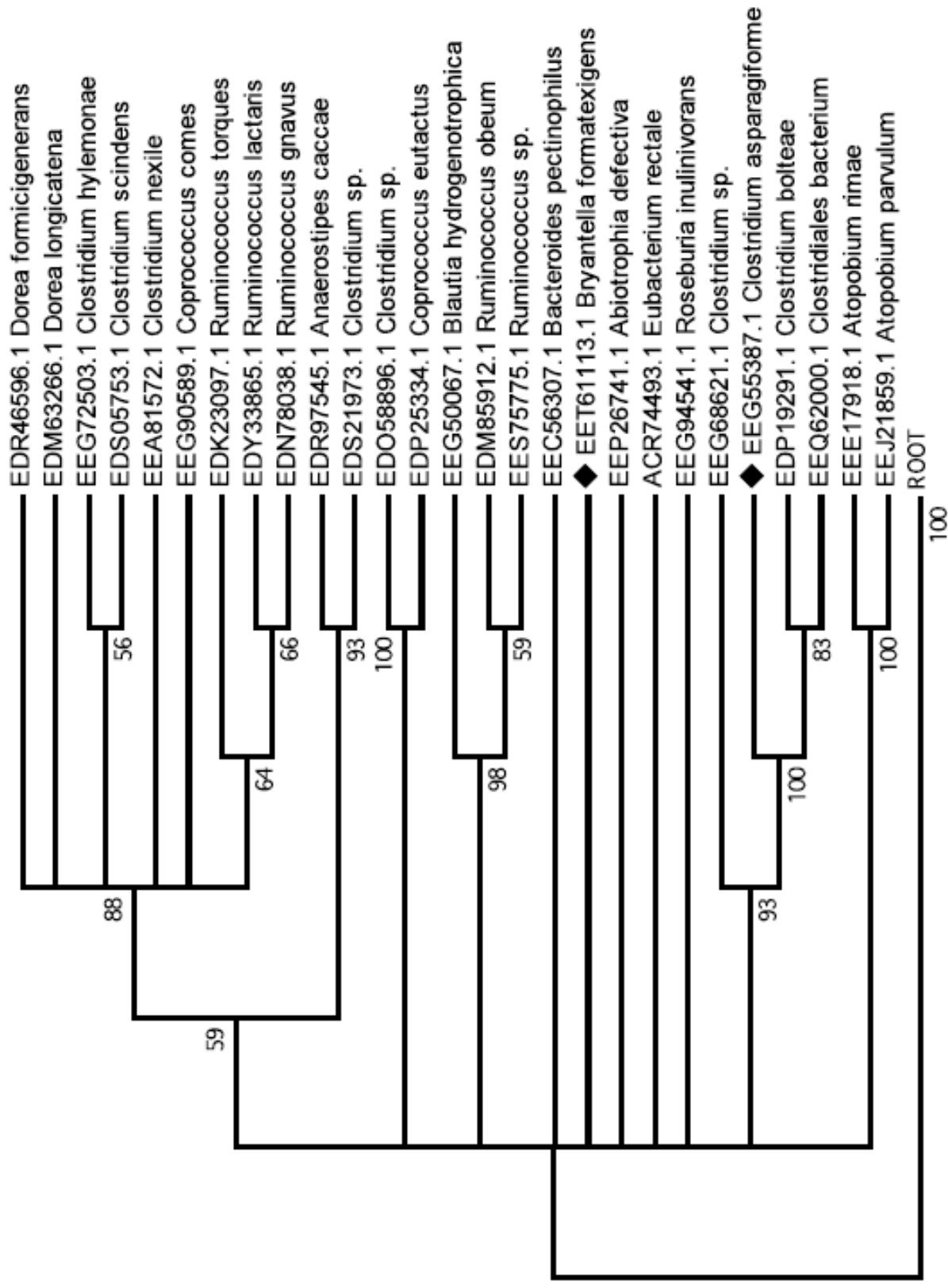

Supplement: Additional file 9 — Figure A8: Rooted phylogenetic tree of Subfamily H. Black diamonds mark genes that were synthesized for functional characterization. [file 1471-2091-12-44-S9.PDF]

Additional file 10, Fig. A9. Subfamily I phylogenetic tree.

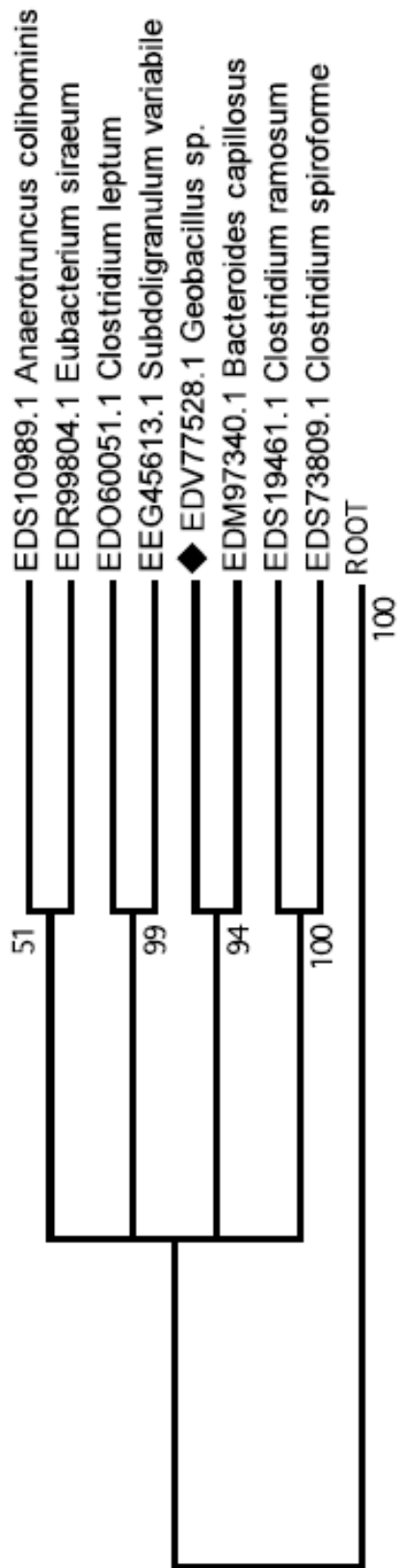

Supplement: Additional file 10 — Figure A9: Rooted phylogenetic tree of Subfamily I. Black diamonds mark genes that were synthesized for functional characterization. [file 1471-2091-12-44-S10.PDF]

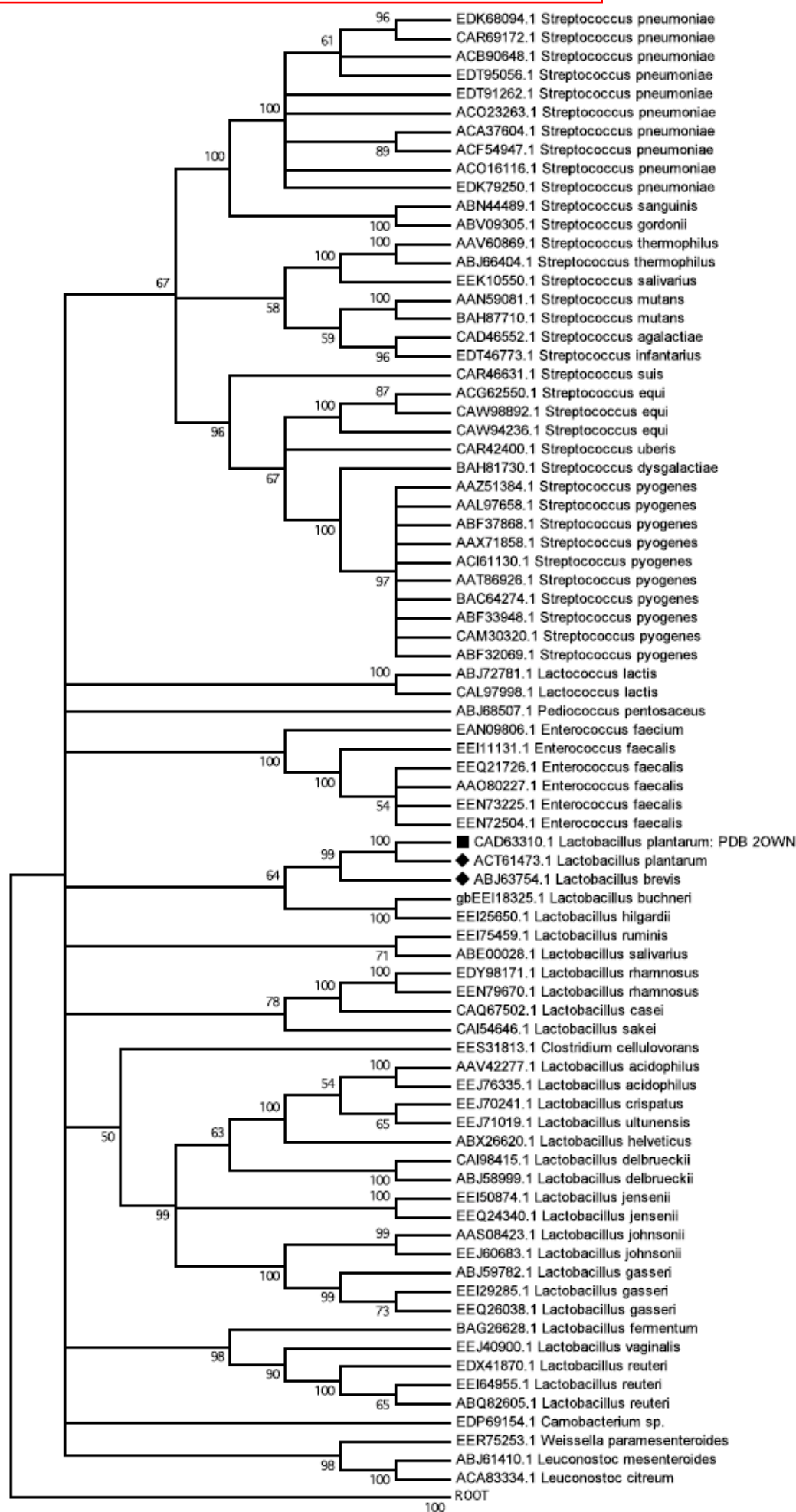

Supplement: Additional file 11 — Figure A10: Rooted phylogenetic tree of Subfamily J. Black diamonds mark genes that were synthesized for functional characterization. [file 1471-2091-12-44-S11.PDF]
