# Supplementary material for: Phylogenetic and experimental characterization of an acyl-ACP thioesterase family reveals significant diversity in enzymatic specificity and activity
Source: BMC Biochem. 2011 Aug 10;12:44. doi: 10.1186/1471-2091-12-44 (PMC3176148; doi:10.1186/1471-2091-12-44)
Supplement: Additional file 4 — Figure A3: Rooted phylogenetic tree of Subfamily C. [file 1471-2091-12-44-S4.PDF]

Additional file 4, Figure A3. Subfamily C phylogenetic tree.

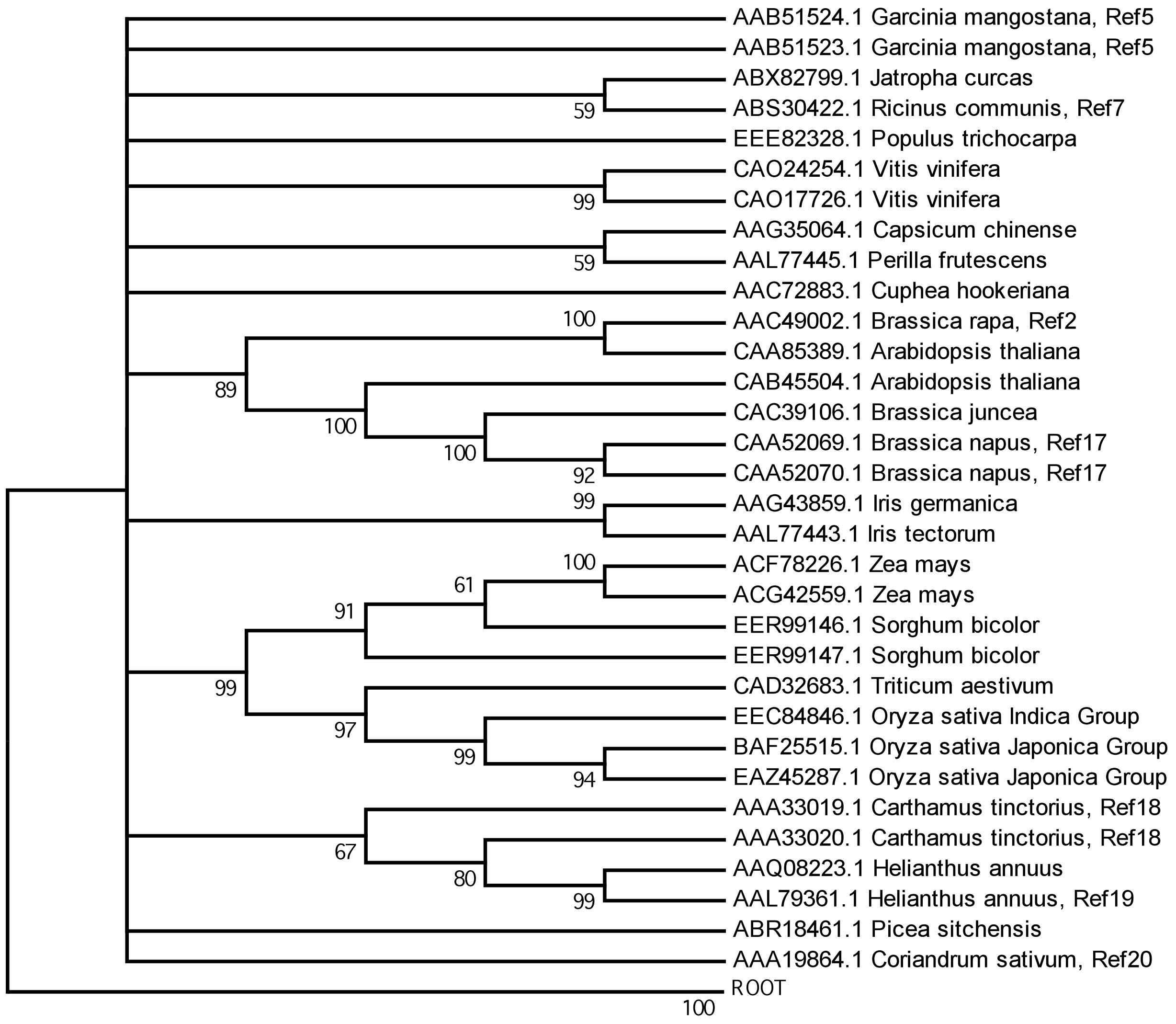

References to Citations Found in Figs. A1 and A3

1. Dormann P, Voelker TA, Ohlrogge JB: **Cloning and expression in *Escherichia coli* of a novel thioesterase from *Arabidopsis thaliana* specific for long-chain acyl-acyl carrier proteins.** *Arch Biochem Biophys* 1995, **316**:612–618.

2. Jones A, Davies HM, Voelker TA: **Palmitoyl-acyl carrier protein (ACP) thioesterase and the evolutionary origin of plant acyl-ACP thioesterases.** *Plant Cell 1995,* **7**:359–371.

3. Yoder DW, Nampaisansuk M, Pirtle IL, Chapman KD, Pirtle RM: **Molecular cloning and nucleotide sequence of a gene encoding a cotton palmitoyl-acyl carrier protein thioesterase.** *Biochim Biophys Acta 1999,* **1446**:403–413.

4. Pirtle RM, Yoder DW, Huynh TT, Nampaisansuk M, Pirtle IL, Chapman KD: **Characterization of a palmitoyl-acyl carrier protein thioesterase (FatB1) in cotton.** *Plant Cell Physiol 1999,* **40**:155–163.

5. Hawkins DJ, Kridl JC: **Characterization of acyl-ACP thioesterases from mangosteen (*Garcinia mangostana*) seed and high level of stearate**. *Plant J 1998,* **13**: 743–752.

6. Wu PZ, Li J, Wei Q, Zeng L, Chen YP, Li MR, Jiang HW, Wu GJ: **Cloning and functional characterization of an acyl-acyl carrier protein thioesterase (JcFatB1) from *Jatropha curcas*.** *Tree Physiol 2009,* **29**:1299–1305.

7. Sanchez-Garcia A, Moreno-Perez AJ, Muro-Pastor AM, Salas JJ, Garces R, Martinez-Force E: **Acyl-ACP thioesterase from castor (*Ricinus communis* L.): an enzymatic system appropiate for high rates of oil synthesis and accumulation.** *Phytochemistry 2010,* **71**:860–869.

8. Zhou Z, Zhang D, Lu M: **Cloning and expression analysis of PtFatB gene encoding the acyl-acyl carrier protein thioesterase in *Populus tomentosa* Carr.** *J Genet Genomics 2007,* **34**:267–273.

9. Jha JK, Maiti MK, Bhattacharjee A, Basu A, Sen PC, Sen SK: **Cloning and functional expression of an acyl-ACP thioesterase FatB type from *Diploknema* (*Madhuca*) *butyracea* seed in *Escherichia coli***. *Plant Physiol Biochem 2006,* **44**:645–655.

10. Ghosh SK, Bhattacharjee A, Jha JK, Mondal AK, Maiti MK, Basu A, Ghosh D, Ghosh S, Sen SK: **Characterization and cloning of a stearoyl/oleoyl specific fatty acyl-acyl carrier protein thioesterase from the seeds of *Madhuca longifoli* (*latifolia*).** *Plant Physiol Biochem 2007,* **45**:887–897.

11. Voelker TA, Jones A, Cranmer AM, Davies HM, Knutzon DS: **Broad-range and binary-range acyl-acyl carrier protein thioesterases suggest an alternative mechanism for medium-chain production in seeds.** *Plant Physiol 1997,* **114**:669–677.

12. Dehesh K, Jones A, Knutzon DS, Voelker TA: **Production of high levels of 8:0 and 10:0 fatty acids in transgenic canola by overexpression of Ch FatB2, a thioesterase cDNA from *Cuphea hookeriana*.** *Plant J 1996,* **9**:167–172.

13. Dehesh K, Edwards P, Hayes T, Cranmer AM, Fillatti J: **Two novel thioesterases are key determinants of the bimodal distribution of acyl chain length of *Cuphea palustris* seed oil.** *Plant Physiol 1996,* **110**:203–210.

14. Leonard JM, Slabaugh MB, Knapp SJ: ***Cuphea wrightii* thioesterases have unexpected broad specificities on saturated fatty acids.** *Plant Mol Biol 1997,* **34**:669–679.

15. Voelker TA, Worrell AC, Anderson L, Bleibaum J, Fan C, Hawkins DJ, Radke SE, Davies HM: **Fatty acid biosynthesis redirected to medium chains in transgenic oilseed plants**. *Science 1992,* **25**:72–74.

16. Yuan L, Voelker TA, Hawkins DJ: **Modification of the substrate specificity of an acyl-acyl carrier protein thioesterase by protein engineering.** *Proc Natl Acad Sci USA 1995,* **92**:10639–10643

17. Loader NM, Woolner EM, Hellyer A, Slabas AR, Safford R: **Isolation and characterization of two *Brassica napus* embryo acyl-ACP thioesterase cDNA clones**. *Plant Mol Biol 1993,* **23**:769–778.

18. Knutzon DS, Bleibaum JL, Nelsen J, Kridl JC, Thompson GA: **Isolation and characterization of two safflower oleoyl-acyl carrier protein thioesterase cDNA clones**. *Plant Physiol 1992,* **100**:1751–1758.

19. Serrano-Vega MJ, Garces R, Martinez-Force E: **Cloning, characterization and structural model of a FatA-type thioesterase from sunflower seeds (*Helianthus annus* L.).** *Planta 2005,* **221**:868–880.

20. Dormann P, Kridl JC, Ohlrogge JB: **Cloning and expression in *Escherichia coli* of a cDNA coding for the oleoyl-acyl carrier protein thioesterase from coriander (*Coriandrum sativum* L.).** *Biochim Biophys Acta 1994,* **1212**:134–136.
